# Design, synthesis, and *in vitro* antiproliferative evaluation of dasatinib/HO-1 inhibitor codrugs towards glioblastoma therapy

**DOI:** 10.1039/d6md00227g

**Published:** 2026-10-07

**Authors:** Valeria Pittalà, Loredana Salerno, Valeria Consoli, Agata Grazia D'Amico, Valeria Sorrenti, Luca Vanella, Giorgia Centamore, Chiara Terlati, Sara Mocka, Maria Nunziata Modica, Giuseppe Romeo, Marica Erminia Schiano, Federica Sodano, Mohamed A. Helal, Sebastiano Intagliata

**Affiliations:** a Department of Drug and Health Sciences, University of Catania viale A. Doria 6 95125 Catania Italy s.intagliata@unict.it +39 095 738 4053; b Department of Pharmacy, “Federico II” University of Naples 80131 Naples Italy; c Biomedical Sciences Program, University of Science and Technology, Zewail City of Science and Technology Giza 12587 Egypt; d Medicinal Chemistry Department, Faculty of Pharmacy, Suez Canal University Ismailia 41522 Egypt

## Abstract

Herein, dasatinib (DASA) and heme oxygenase-1 (HO-1) inhibitor codrugs were designed and synthesized. Derivatization of DASA was performed by preserving the structural features essential for target inhibition and was rationally guided by molecular modelling studies. Two out of three synthesized codrugs showed the ability to inhibit HO-1 activity in a low micromolar range (*i.e.*, 2.12 and 1.00 μM for TC7 and TC8, respectively), while all derivatives retained a significant capability of inhibiting the cSRC tyrosine kinase with sub-micromolar potency (*i.e.*, 0.0023–0.0255 μM). Of note, derivative TC7 exhibited significant *in vitro* antiproliferative activity against U87MG glioblastoma cells, likely mediated by HO inhibition and accompanied by a ∼1.5-fold increase in intracellular ROS levels compared with the control. Also, assessment of the most promising codrug, TC7, revealed high chemical and enzymatic stability (*t*_1/2_ >24 h). While additional optimization will be required to further improve its drug-like profile, TC7 emerged as the most promising compound evaluated in this study and represents a suitable starting point for future development. Collectively, these results serve as a proof of concept supporting a potential new codrugs strategy combining HO-1 and cSRC inhibition for glioblastoma therapy.

## Abstract 2

Novel dasatinib and heme oxygenase-1 inhibitor codrugs were developed as a promising dual-target strategy combining HO-1 and c-Src inhibition for glioblastoma therapy.

## Graphical abstract

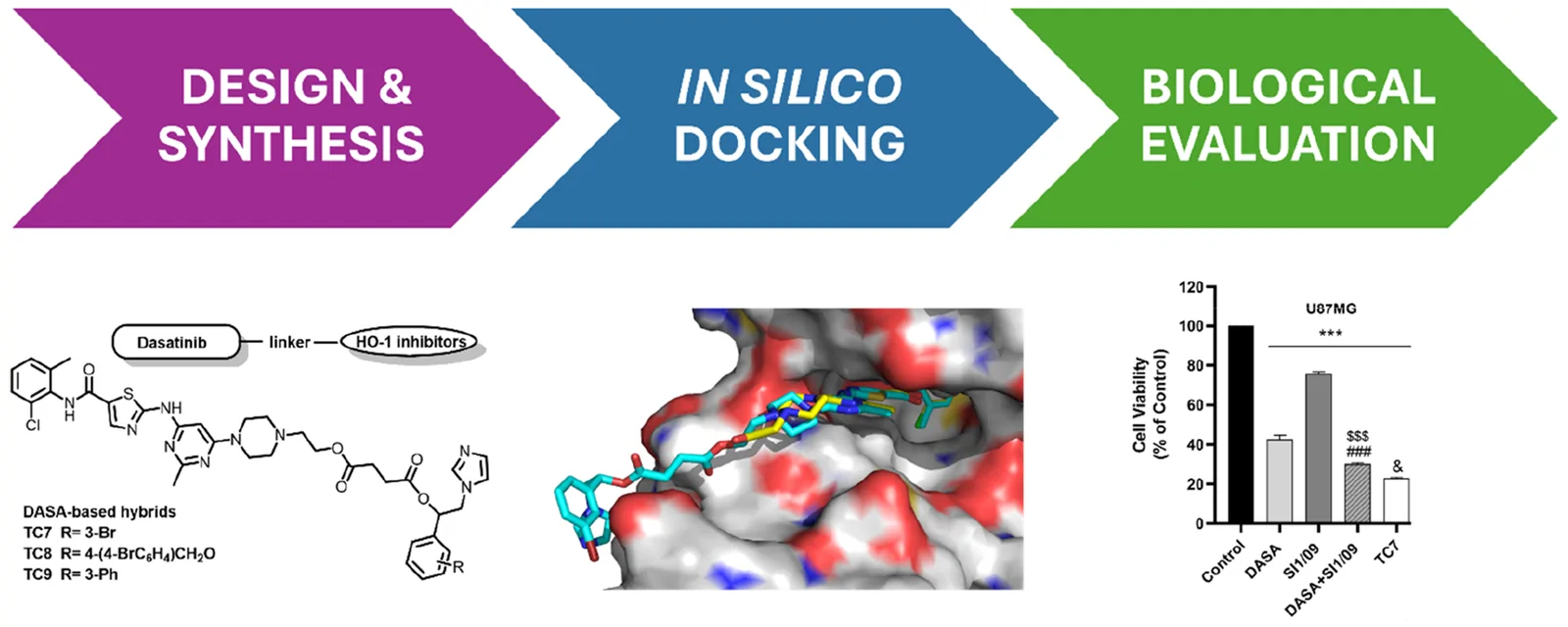

## Introduction

Glioblastoma, also known as glioblastoma multiforme (GBM), is one of the most common and aggressive primary brain tumors in adults. Despite advances in GBM treatment modalities, this malignancy remains largely incurable. Also, it is associated with significant neurological dysfunction (*e.g.*, impairment of thinking and cognition), which severely affects patients' quality of life and increases the overall disease burden. Currently, the gold standard treatment for GBM includes surgical resection followed by radiation therapy and chemotherapy (chemoradiation). Although the most commonly used drug is the alkylating agent temozolomide (TMZ, Temodal®), its efficacy as systemic monotherapy after surgery is limited. More recently, a variety of small-molecule inhibitors have been explored as additional therapeutic options to improve patient survival, including the tyrosine kinase inhibitor (TKI) dasatinib (DASA, BMS-354825, Sprycel®), which has also been evaluated in a phase II trial in patients with recurrent GBM (RTOG 0627).^1^ Surprisingly, unlike promising preclinical results, no satisfactory clinical outcomes have been achieved. Therefore, GBM treatment largely remains an unmet medical need, and the development of safer, more efficacious, and orally available drugs remains crucial. Given the extremely heterogeneous nature and molecular complexity of cancer, the pharmacological modulation of multiple molecular pathways has been proposed as a potential strategy compared to a monotherapy approach to effectively tackle GBM.^2^ Accordingly, the identification of additional biochemical pathways that can be modulated simultaneously with a primary one may be pivotal for enhancing the therapeutic efficacy of existing drugs through additive or synergistic effects. In this context, heme oxygenase-1 (HO-1) has emerged as a potential target in cancer therapy and, specifically, in GBM.^3^ HO-1 is an inducible isoform belonging to the heat shock protein family and is responsible for the catabolism of the heme group.^4,5^ HO-1 is mainly expressed in the liver and spleen and is overexpressed in many cancer cell types. Elevated expression levels of this enzyme have often been linked to tumor growth, aggressiveness, and drug resistance, supporting the development of HO-1 inhibitors as a new tool in cancer chemotherapy.^6^ Interestingly, HO-1 overexpression has been detected in human GBM tumors, and increased enzyme activity has been associated with tumor growth and progression, thereby adversely affecting prognosis in patients with GBM.^7–9^ Indeed, a growing body of evidence suggests that HO-1 may represent a potential therapeutic target in this cancer type;^10^ in particular, non-competitive HO-1 inhibitors have been proposed as chemosensitizer agents in adjuvant cancer chemotherapy.^11–13^ With this in mind, this work aimed to develop novel DASA/HO-1 inhibitor codrugs as dual target modulators towards GBM (*i.e.*, cSRC and HO-1). The codrug approach (*i.e.*, two pharmacologically active molecules covalently combined into a single chemical entity)^14^ was selected to integrate the biological activities of DASA and HO-1 inhibitors into a single molecule, providing an attractive strategy to explore dual-target modulation as an alternative to the administration of the individual agents in combination.

To the best of our knowledge, such a strategy combining an HO-1 inhibitor with the well-known TKI DASA has not yet been reported in the context of GBM treatment, thus providing proof of concept.

## Results and discussion

### Rational design

Novel codrugs, named TC7, TC8, and TC9, were obtained by conjugating DASA with our previously discovered potent imidazole-based HO-1 inhibitors (*i.e.*, SI 1/09, LS 6/42, or LS 4/28)^15^ through a succinate linker (Fig. 1A). The succinate linker was selected because it can be readily introduced through straightforward synthetic procedures and provides a short, biocompatible moiety capable of connecting the two pharmacophores *via* hydrolysable diester bonds. In addition, succinate has been widely employed in prodrug and codrug design owing to its versatility and suitability for the preparation of ester-based conjugates.^16^ Accordingly, double esterification of both the primary and the secondary hydroxy groups of DASA and aryl ethanol imidazoles, respectively, was considered by using a succinic linker. It is worth noting that successful functionalization of these hydroxy groups with succinate linker without affecting the interactions with the corresponding target (*i.e.*, cSRC and HO-1) has been reported.^17–24^ Hence, the derivatization of DASA was performed by preserving the structural feature that allows tyrosine kinase inhibition. Given the proof-of-concept nature of this work, a small set of three codrugs was designed and synthesized to provide an initial assessment of whether the simultaneous modulation of cSRC and HO-1 through a single molecular entity could represent a viable strategy for glioblastoma treatment.

**Fig. 1:**
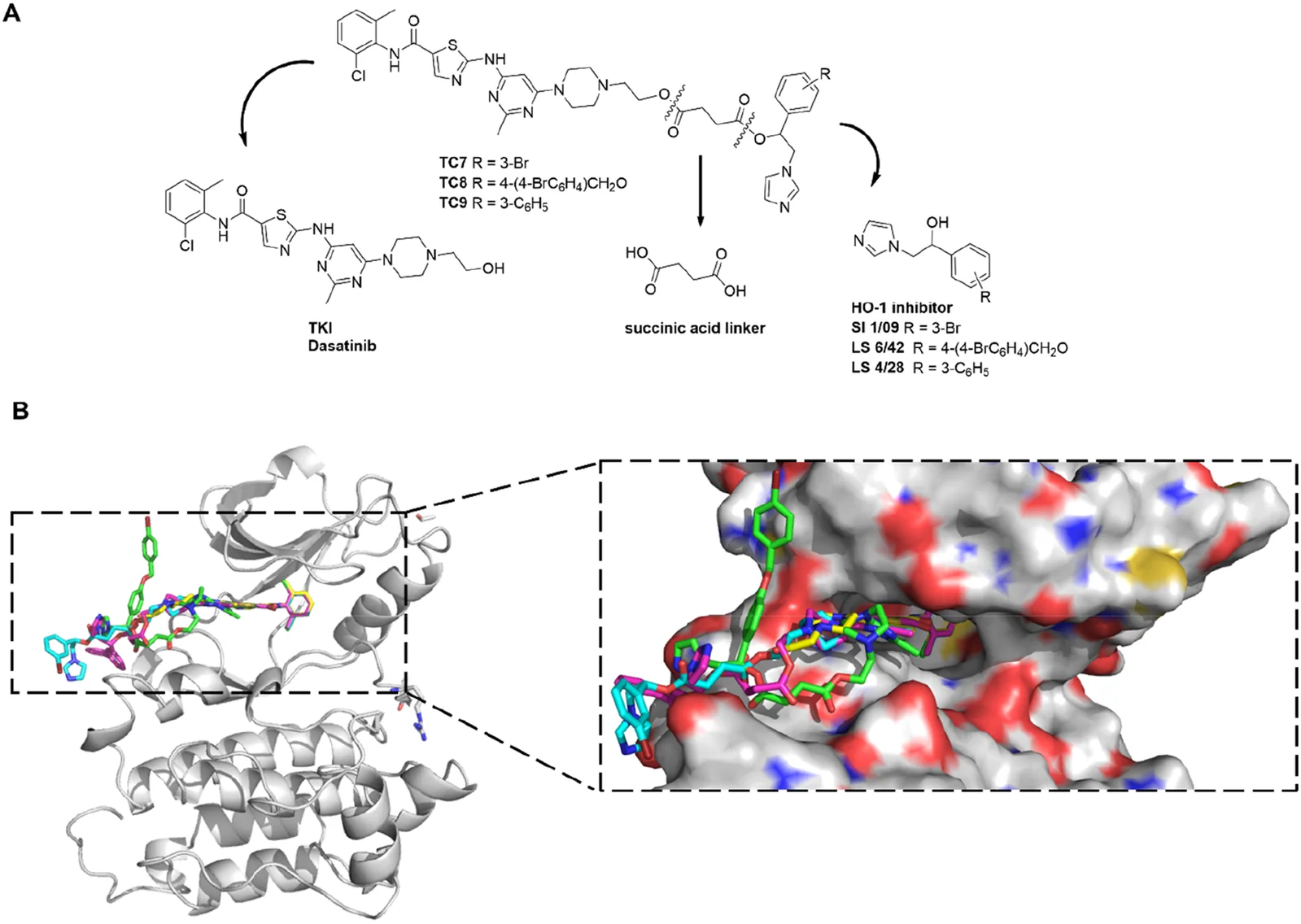
(A) Design strategies and chemical structures of parent drugs and general structure of newly synthesized DASA/HO-1 inhibitor codrugs; (B) the docking simulation using the crystal structure of cSRC (PDB ID 3G5D) demonstrates how codrugs (TC7, cyan; TC8, green; and TC9, magenta) are docked and are superimposed with the co-crystallized DASA (yellow) into the catalytic site.

To corroborate our hypothesis, we first performed molecular modeling simulations by docking the structures of the designed codrugs (TC7, TC8, and TC9) into the cSRC kinase obtained from the available crystal structure (PDB ID 3G5D) (Fig. 1B). As shown in Fig. 1B, our *in silico* predictions supported the rational design of the new compounds since the molecules showed acceptable overlap with the co-crystallized ligand (RMSD = 3.92, 4.08, and 3.85 Å for TC7, TC8, and TC9, respectively). Also, binding energies of docked compounds suggested a favorable interaction of these molecules with the tyrosine kinase target, thus preserving the capability of the part to interact with the ATP binding pocket.

### Chemical synthesis

Reagents and conditions for preparing the final compounds TC7, TC8, and TC9 are summarized in Scheme 1. The synthetic procedure involved a straightforward two-step sequence of reactions. The free primary hydroxyl group of DASA was derivatized with a succinic group *via* reaction with succinic anhydride in the presence of triethylamine (TEA) as a base to promote ring opening. Subsequently, the final DASA/HO-1 inhibitor codrugs TC7, TC8, and TC9 were synthesized *via* esterification of DASA-succ and the appropriate aryl ethanol imidazole SI 1/09, LS 6/42, or LS 4/28, using EDC·HCl as a coupling reagent and DMAP as a catalyst, affording the target compounds in modest yields (20–36%), likely due to the poor solubility of the starting materials in the reaction solvent, which adversely affected reaction efficiency and contributed to product losses during purification.

**Scheme 1:**
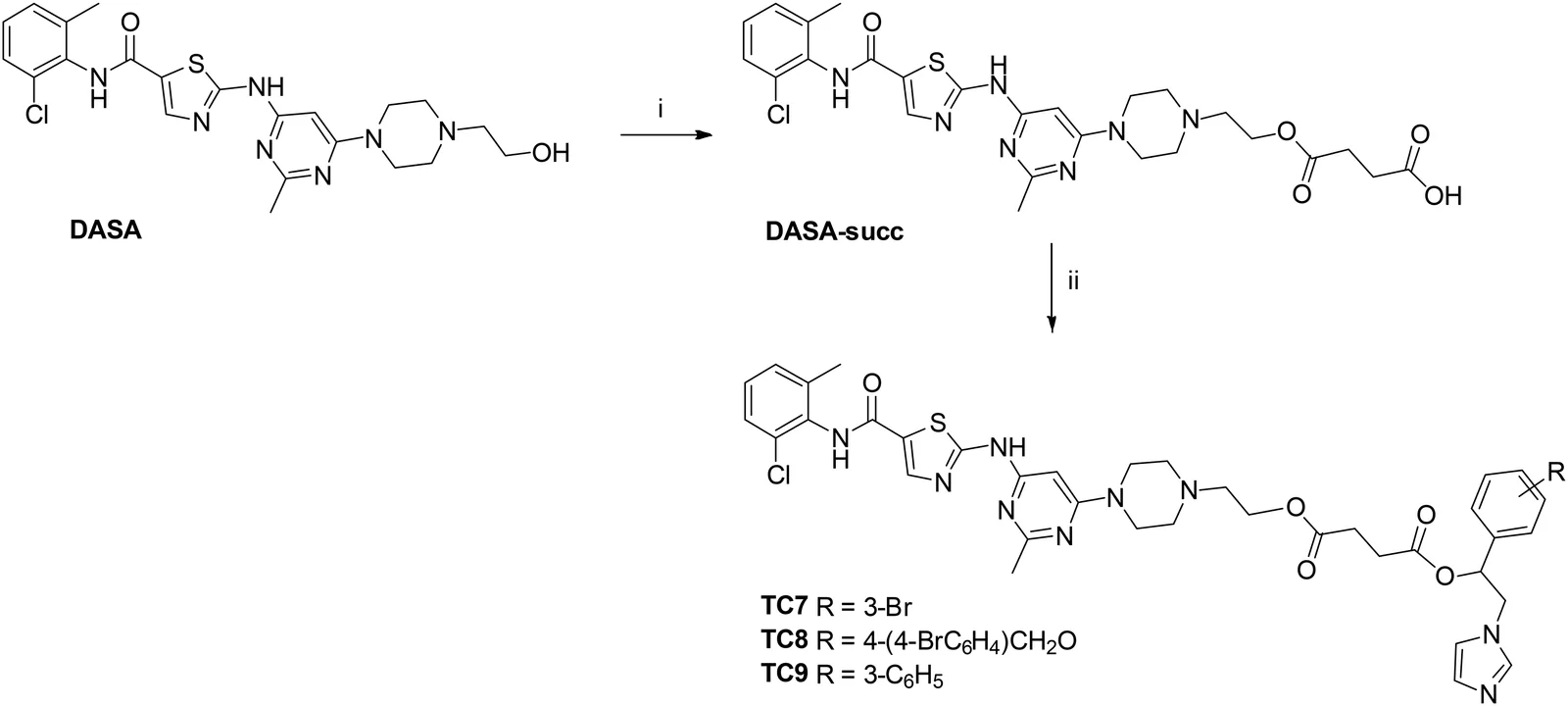
Reagents and conditions: (i) succinic anhydride, TEA, DMF, 60 °C, 24 h, 52%; (ii) aryl ethanol imidazole SI 1/09, LS 6/42, or LS 4/28, EDC·HCl, DMAP, DMF or DMF/DCM (3 : 1 v/v), r.t., 24 h, 20–36%.

^1^H NMR spectral analysis confirmed the structure of the final compounds (SI), as evidenced by the presence of characteristic aromatic signals from the DASA moiety and the absence of the triplet signal at *δ* 4.47 ppm corresponding to the primary alcohol group of parent DASA, indicating successful esterification of DASA-succ (SI, Fig. S1). Final compounds TC7, TC8, and TC9 showed characteristic peaks attributable to the azole derivative, with minor variations in chemical shifts among analogues. For example, in the representative case of TC7, two singlets at *δ* 7.11 and 6.85 ppm correspond to the imidazole protons, while the triplet at *δ* 5.97 ppm and the multiplet at *δ* 4.38 ppm were assigned to methine and methylene groups, respectively (SI, Fig. S4). Ultimately, succinyl protons were detected as a symmetric multiplet at *δ* 2.69–2.54 ppm.

### *In vitro* HO-1/cSRC inhibition

The potency of the newly synthesized compounds to inhibit HO-1 activity was evaluated in a rat spleen microsomal fraction using our well-validated *in vitro* assay.^23^ Enzyme inhibitory potency for each compound is expressed as IC_50_ (μM) and is reported in Table 1. Notably, TC7 and TC8 retained significant HO-1 inhibitory potency, showing activities in a similar range to those of the parent inhibitors SI 1/09 and LS 6/42, respectively, with TC7 displaying an approximately 5-fold reduction in potency compared with SI 1/09. In contrast, the intermediate DASA-succ and derivative TC9 were essentially inactive (Table 1). In addition, the inhibition of cSRC by new codrugs was assessed, revealing a 49-, 64-, and 6-fold reduction in potency for TC7, TC8, and TC9, respectively, compared with DASA. These data are consistent with our previously reported findings on TKIs/HO-1 inhibitor hybrids.^21,22^ Importantly, the observed loss of potency for the newly synthesized compounds is not considered detrimental in the context of a codrug approach, since biotransformation is expected to regenerate the parent molecules responsible for target modulation.

**Table 1:** Inhibitory potency of codrugs and parent drugs towards HO-1 and cSRC

Compound	HO-1 IC_50_ (μM) ± SD^a^	cSRC IC_50_ (μM) ± SD^b^
TC7	2.12 ± 0.03	0.0196 ± 0.005
TC8	1.00 ± 0.05	0.0255 ± 0.007
TC9	>100	0.0023 ± 0.001
SI 1/09^c^	0.40 ± 0.01	—
LS 6/42^c^	0.95 ± 0.02	—
LS 4/28^c^	0.90 ± 0.08	—
DASA-succ	>100	—
DASA^d^	—	0.0004 ± 0.0002

a Values represent the mean ± standard deviation (SD) of triplicate tests. Hemin concentration in the assay was 25 μM (corresponding to 10-fold the reported *K*_m_ value for HO-1).

b Kinase inhibition assays were performed at Eurofins. ATP concentration was at cSRC *K*_m_ value (200 μM). Catalogue number: 14-326KP.

c Data from ref. 15.

d Data from ref. 25.

### Docking studies

Molecular docking simulations were performed to investigate the interactions of codrugs with cSRC kinase and HO-1. The predicted binding modes were analyzed in comparison with co-crystallized DASA bound to cSRC kinase and the azole-based derivative (2*R*,4*S*)-2-(2-(4-chlorophenyl)ethyl)-2-((1*H*-imidazol-1-yl)methyl)-4-(((5-trifluoromethylpyridin-2-yl)thio)methyl)-1,3-dioxolane (QC-80) to HO-1 by using the available X-ray crystal structures of both proteins (*i.e.*, PDB ID 3G5D for cSRC/DASA and PDB ID 3HOK for HO-1/QC-80).^25,26^ Selected docked poses of the codrugs in the HO-1 and cSRC catalytic domains are depicted in Fig. 2 and 3, respectively.

**Fig. 2:**
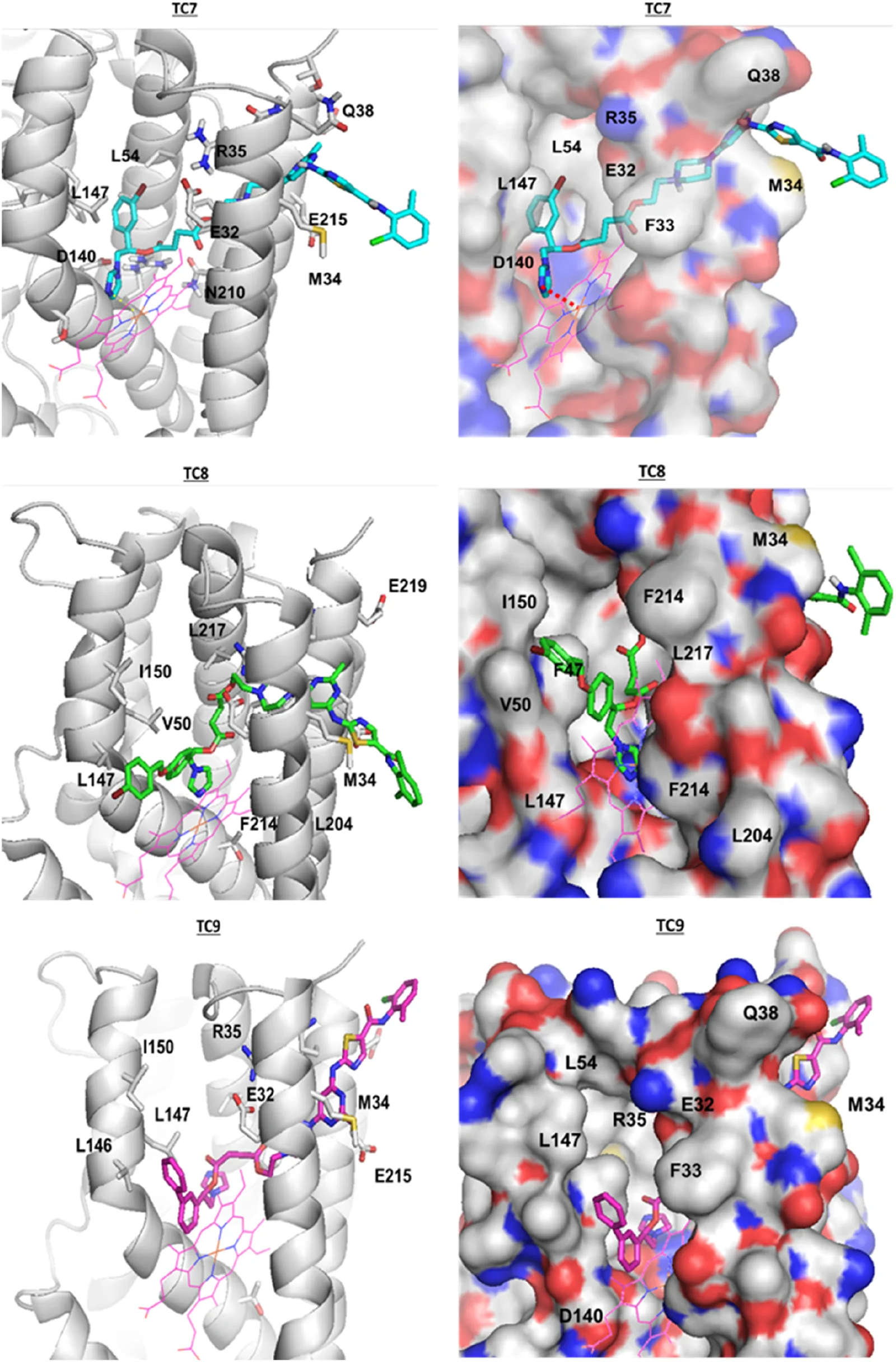
Docking simulation for new DASA/HO-1 inhibitor codrugs towards HO-1 crystal structure. Protein is shown as cartoon representation (left) and as molecular surface (right). Surface colouring represents electrostatic potential (red, negative; blue, positive; white, neutral/hydrophobic regions). Ligands are shown in stick representation (cyan for TC7, green for TC8, and magenta for TC9). The displayed region corresponds to the heme-binding pocket (active site) of HO-1 (PDB ID 3HOK).

**Fig. 3:**
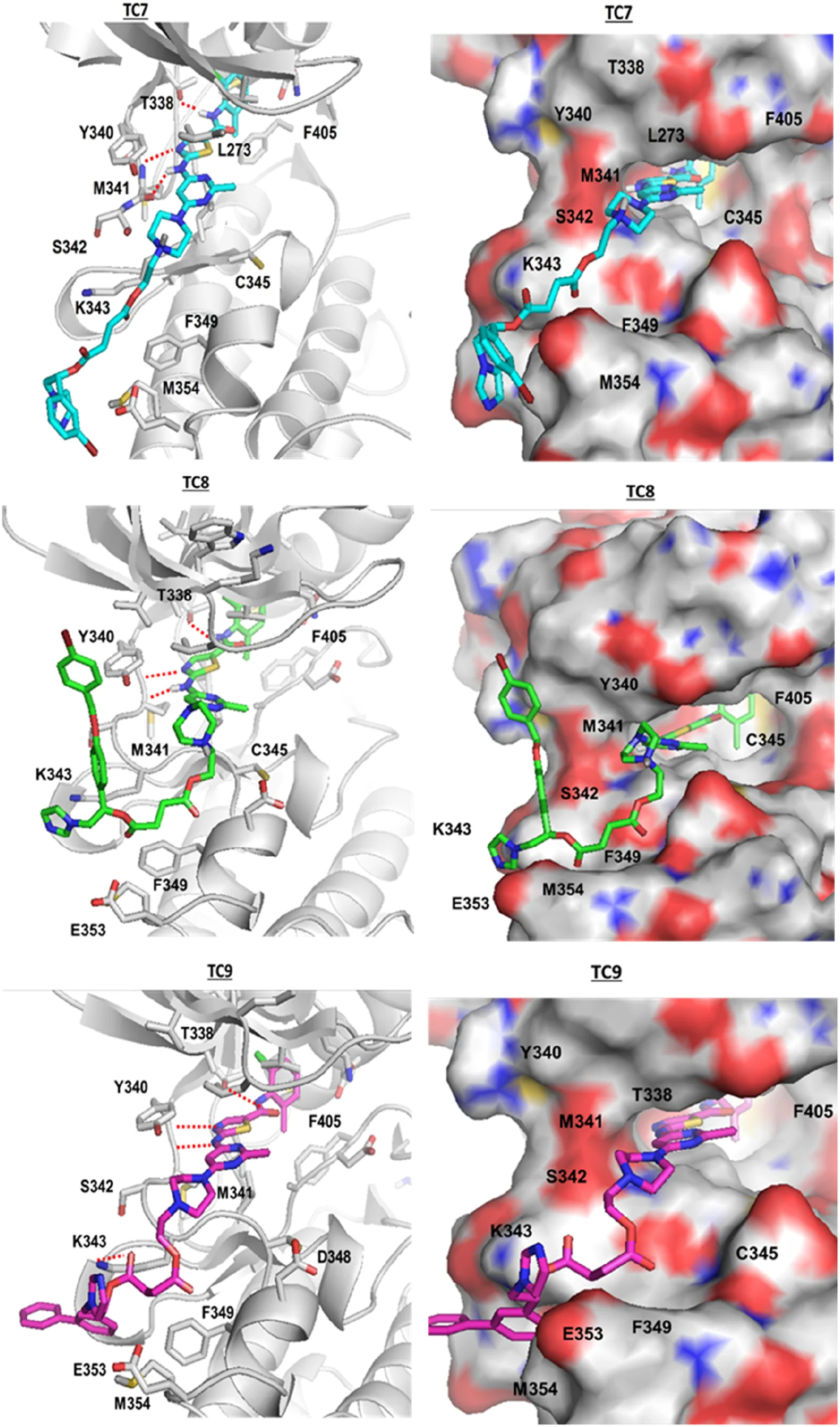
Docking simulation for new DASA/HO-1 inhibitor codrugs towards cSRC crystal structure. Protein is shown as cartoon representation (left) and as molecular surface (right). Surface colouring represents electrostatic potential (red, negative; blue, positive; white, neutral/hydrophobic regions). Ligands are shown in stick representation (cyan for TC7, green for TC8, and magenta for TC9). The displayed region corresponds to the ATP-binding site within the catalytic domain of c-SRC (PDB ID 3G5D).

In the crystal structures of imidazole-based HO-1 inhibitors, the ligands coordinate the heme iron *via* the imidazole nitrogen, while a relatively small aromatic/hydrophobic ring fits in a distal hydrophobic pocket formed by Phe47, Val50, Leu54, and Ile147. Notably, in the PDB 3HOK structure used here, the ligand QC-80 is bulkier, with a substituted pyridine installed on the dioxolane ring, providing a Y-shaped structure.

The binding of bulkier inhibitors entails expansion of the heme-binding pocket to accommodate the ligand without displacement of the heme moiety. This is proposed to occur *via* outward movement of the flexible distal helix of human HO-1, creating space for the third arm of the inhibitor, corresponding to the DASA part in our designed molecules (Fig. 2). It is worth noting that the HO-1 crystal structure used in this docking study is already showing the induced binding mode with a relatively bulky inhibitor bound in the hydrophobic pocket created after a significant shift in the proximal helix. The software used, FRED, is a rigid docking algorithm regarding the protein. However, it uses a large conformer library of the ligand, generated prior to the docking process. Docking poses with the highest scores were selected, prioritizing those placing the imidazole ring in the heme binding site (SI, Table S1). It was noticed that the docking poses of the molecules TC7 to TC9 overlap, with various degrees, with the crystallized imidazole–dioxolane ligand, approaching the heme and the critical Asp140, which is normally involved in an ordered solvent structure with the heme iron. As expected, TC7 showed the best overlap with its imidazole nitrogen at position 3 coordinating with the heme iron, consistent with a hexacoordinated heme. The 3-bromophenyl ring fits nicely into the aforementioned distal hydrophobic pocket, while the DASA arm extends outwards behind the flexible distal helix. For TC8, which contains a bulkier 4-bromobenzyloxy substituent, the two hydrophobic arms of the ligand were inverted to allow the bulky benzyloxy group to exit above the heme while maintaining the imidazole ring close enough to the heme iron for potential water-mediated interactions. When this inversion happens in compound TC9, it causes the imidazole ring to point upwards away from the heme iron, preventing any possible coordination with the iron center. This is due to the *meta*-substituted phenyl arm, which requires a slight rotation of the ligand to avoid any steric clashes with the residues above the heme, such as Val146 or Ile150. Although TC8 is bulkier, the presence of the large 4-bromobenzyloxy moiety on the *para* position of the phenyl ring allows its protrusion towards the solvent without any clashes. This could rationalize, at least in part, the loss of HO-1 inhibitory activity for TC9.

On the other hand, docking into the ATP-binding site of the cSRC kinase was more straightforward. All compounds, TC7 to TC9, adopt the typical type II inhibitor orientation of DASA, exposing the imidazole terminal to the solvent (Fig. 3).^25^ As expected, the central NH and the thiazole nitrogen of the DASA fragment form hydrogen bonds with the hinge residue M341. In addition, the aniline moiety probes deeply into the hydrophobic back pocket with its NH forming another H-bond with the gatekeeper Thr338. The variable portion of the compounds is solvent-exposed, showing hydrophobic contact with the enzyme surface. Notably, TC9 achieves the greatest surface burial at this solvent-exposed site, which may contribute to its relatively higher affinity for cSRC compared to TC7 and TC8.

### *In vitro* antiproliferative activity

The antiproliferative effect of newly synthesized DASA/HO-1 inhibitor codrugs and reference compounds was evaluated in the GBM cell line U87MG. Data shown in Fig. 4 indicate that all three compounds maintained a good potency compared to DASA.

**Fig. 4:**
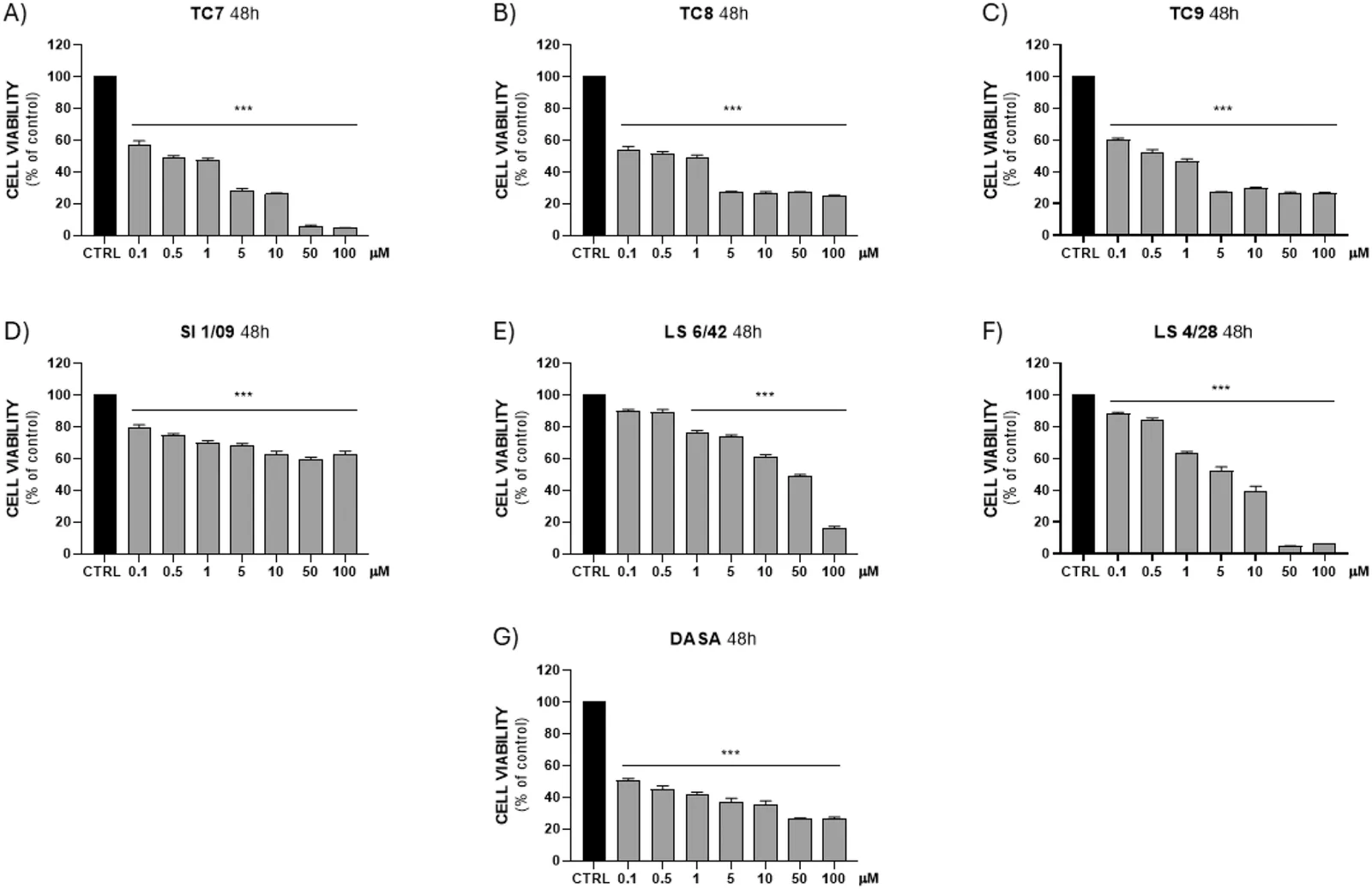
Evaluation of the effect of newly synthesized hybrids TC7, TC8, and TC9 (A–C), HO-1 inhibitors (D–F) and DASA (G) at different concentrations on GBM U87MG cell line viability following 48 h of treatment. Data are presented as mean ± SEM (***p < 0.001 *vs.* control).

In addition, codrugs and the corresponding parent drugs administered in a 1 : 1 combination were evaluated at a fixed dose to compare the ability of codrugs to mimic the effect of the drug combination (Fig. 5). While this approach enables a direct comparison of codrugs *vs.* combo treatment, it lacks combination-index analyses, preventing quantitative assessment of combination effects. Nevertheless, codrugs showed a significant antiproliferative effect in U87MG cells, generally greater than that of the parent compounds at the tested concentration. Notably, TC7 stood out as the best compound, producing a statistically significant reduction in cell viability compared to both control and combination treatment. Additionally, TC7, the parent compounds, and the corresponding combination treatment were evaluated in a second GBM cell line, A172, to determine whether their antiproliferative activity was maintained in a cellular context characterized by lower basal HO-1 expression levels (SI, Fig. S13). Based on previously published data,^8^ U87MG cells were selected as the primary model for codrug evaluation because of their relatively elevated basal HO-1 expression compared with A172 cells. Consistent with our hypothesis, neither the HO-1 inhibitor SI 1/09 nor the TC7 hybrid exerted a significant effect on A172 cell viability under the same experimental conditions, whereas DASA displayed a comparable antiproliferative effect in both cell lines. These findings suggest that the biological activity associated with HO-1 targeting may be influenced by the basal expression status of the enzyme and support the rationale for selecting HO-1-high cellular models to maximize the efficacy of HO-1-directed strategies. At the same time, the retained sensitivity to DASA in A172 cells indicates that its cytotoxic activity is not dependent on HO-1 modulation and involves additional mechanisms of action. Accordingly, TC7 was selected for further *in vitro* investigations.

**Fig. 5:**
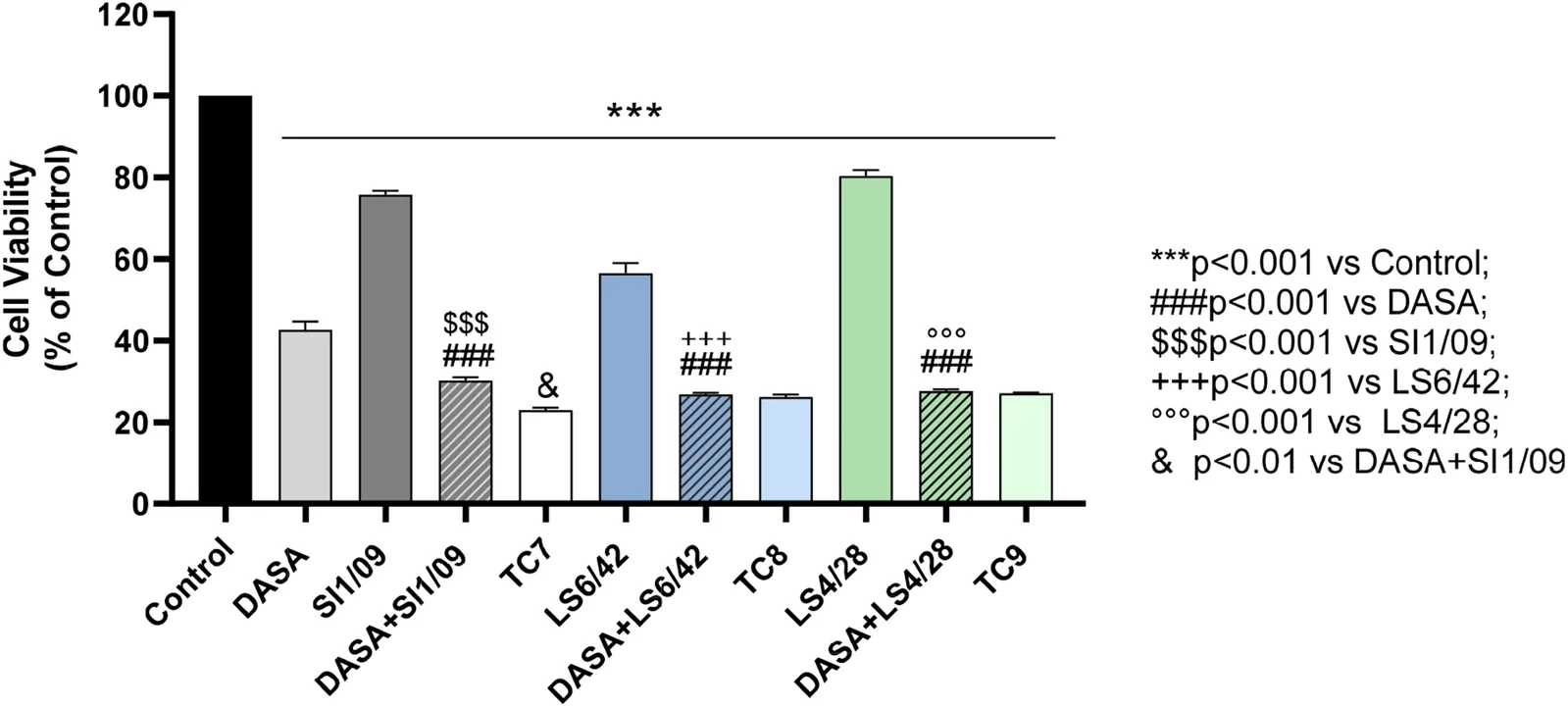
Effect of DASA, HO-1 inhibitors, combo, and codrug treatments (5 μM) on U87MG cell viability at 48 h. Data are presented as mean ± SEM. Statistical significances are reported in the figure.

### Effect of TC7 on HO-1 inhibition and reactive oxygen species (ROS) induction in U87MG cells

To investigate the relationship between HO-1 modulation and the antiproliferative sensitization of U87MG cells to DASA, the inhibitory activity of TC7 was tested in cell lysates. As shown in Fig. 6A, TC7 induced a slight reduction in HO activity in U87MG cell lysates; however, this effect was less pronounced than that observed with the reference HO-1 inhibitor SI 1/09, likely due to a compensatory upregulation of HO-1 activity induced by DASA (Fig. 6A). Indeed, evaluation of HO-1 protein expression showed a remarkable increase in response to DASA treatment (of approximately 7-fold) accompanied by no relevant changes after SI 1/09 treatment and a modest increase in the case of TC7 (Fig. 6B), which is consistent with non-observed significant enzymatic inhibition. Notably, TC7 elicited a significant increase in intracellular ROS levels in U87MG cells, exceeding those induced by SI 1/09 and comparable to those observed with the combined treatment (Fig. 6C). Further studies will be required to elucidate the molecular mechanisms underlying this effect. Additionally, quantification of thiols at the intracellular levels, which reflect GSH content (Fig. 6D), displayed once again an increase following DASA treatment, suggesting activation of antioxidant adaptive responses independently of direct HO-1 inhibition, even if there was no appreciable ROS induction. This observation suggests that DASA may stimulate cytoprotective or redox-regulatory pathways before overt oxidative stress becomes detectable. These findings are in agreement with previous reports describing a context-dependent mechanism underlying DASA-induced cytotoxicity,^27–29^ where cellular outcome appears to depend on the balance between oxidative stress induction and activation of compensatory antioxidant responses.

**Fig. 6:**
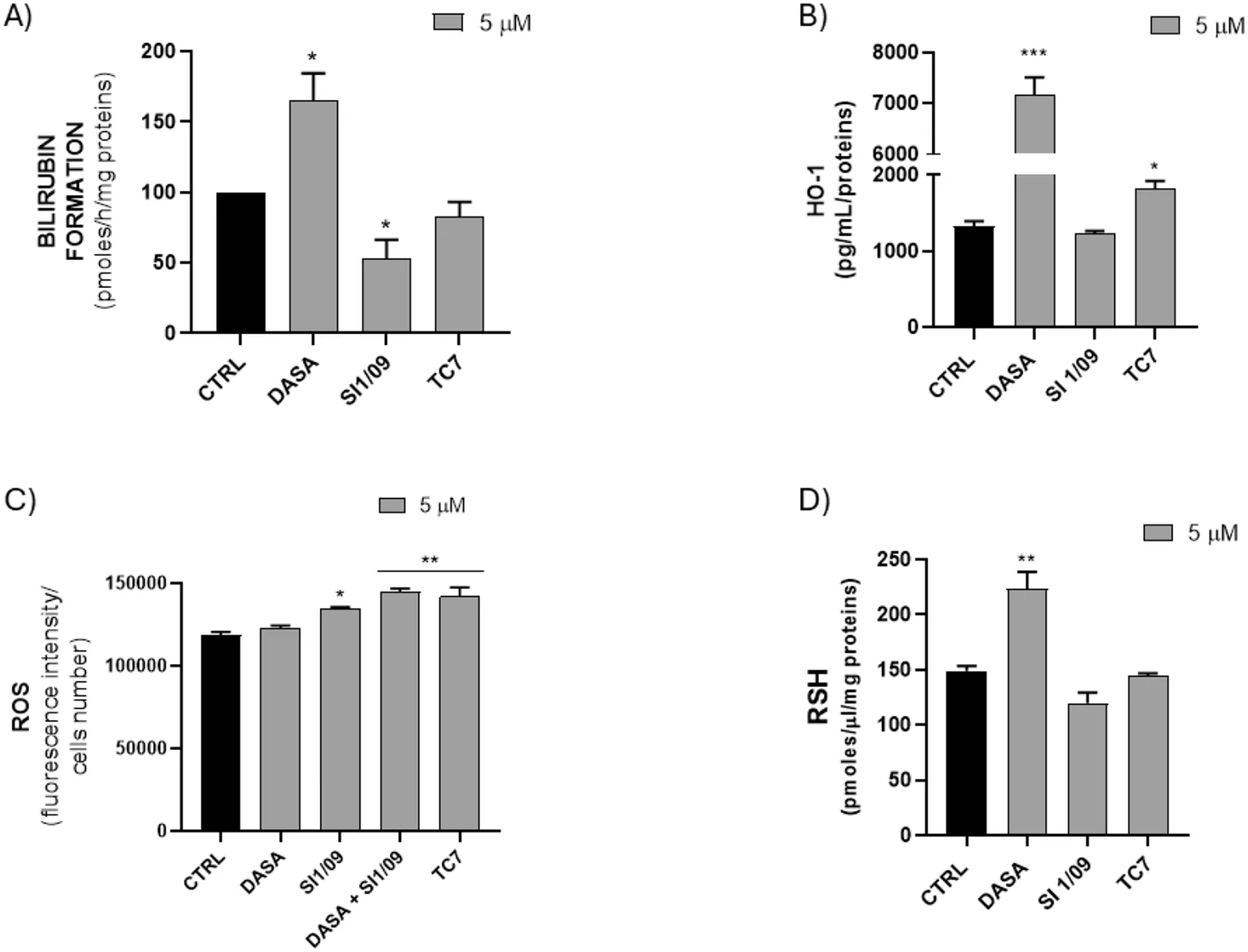
(A and B) Evaluation of HO-1 enzymatic activity and protein expression levels in untreated (CTRL) and treated (*i.e.*, 5 μM DASA, SI 1/09, and TC7) U87MG cells. (C) Evaluation of ROS level increase in U87MG cells after treatment with DASA, HO-1 inhibitors, combo, and codrugs at fixed concentrations (5 μM). (D) Measurement of intracellular thiol group (RSH) concentration following treatments (*p < 0.05, **p < 0.005, ***p < 0.0005 *vs.* CTRL).

Conversely, treatment with the HO-1 inhibitor SI 1/09 resulted in a slight reduction in intracellular thiol levels, while no significant changes were observed for TC7. Considering the concomitant increase in ROS production, this behaviour may reflect enhanced consumption of intracellular glutathione to buffer oxidative stress. Taken together, these results suggest that modulation of HO-1 contributes more prominently to the pro-oxidant effects associated with TC7 treatment and may represent an important determinant of its cytotoxic activity. Nevertheless, this interpretation remains putative and would benefit from further mechanistic validation.

### Chemical and enzymatic stability of TC7

The chemical and enzymatic stability of TC7 was evaluated at physiological temperature (37 °C) to simulate human body conditions. Two incubation media were employed: phosphate-buffered saline (PBS) to assess stability at physiological pH and to characterize the degradation profile, and esterase-rich Dulbecco's modified Eagle medium (DMEM) to mimic the enzymatic environment of biological assays and predict the codrug's behavior under experimental conditions. As shown in Fig. 7A, TC7 was highly stable in PBS, with negligible degradation (<5%) after 24 h of incubation, indicating an effectively long half-life under these conditions (SI, Fig. S14). In DMEM (Fig. 7B), the presence of enzymatic pathways increased the degradation rate, yet the percentage of degraded compound remained below 50% after 24 h. The calculated half-life in this medium was 24.8 h, with the degradation profile monitored up to 26 h (SI, Fig. S15). Importantly, the main metabolite detected during degradation corresponded to the parent drug, DASA, confirming that hydrolysis occurs without unexpected side products. Overall, these results indicate that TC7 exhibits both chemical and enzymatic stability compatible with biological testing. Furthermore, the molecular hybridization approach not only provides biological advantages but also confers favorable physicochemical properties, contributing to the stability of the compound in physiological and enzymatic environments.

**Fig. 7:**
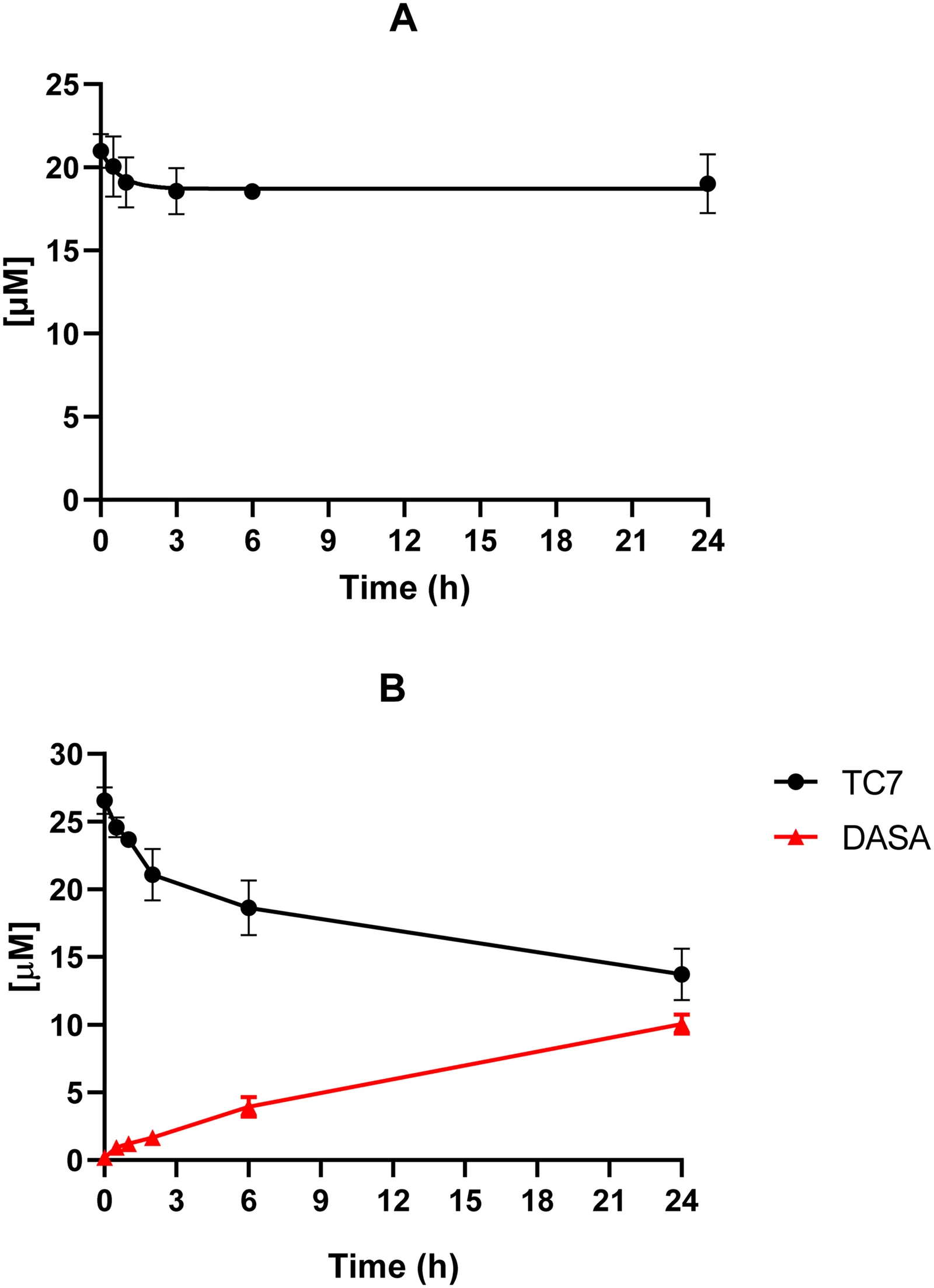
Chemical stability of TC7 in PBS (A) and enzymatic stability in DMEM cell culture medium (B). Data are expressed as mean ± SD of the concentrations of unmodified codrug and released parent compound (DASA) over time, measured in μM.

### *In vitro* ADMET assessment

A preliminary evaluation of the *in vitro* ADME properties (*i.e.*, aqueous solubility, partition coefficient, metabolic stability) and off-target toxicity (*i.e.*, binding towards hERG potassium channel) was performed for compound TC7 (Table 2).

**Table 2:** *In vitro* ADMET profile of TC7

Test type	TC7	Reference cmpd.
Aqueous solubility (PBS, pH 7.4)^a^	0.50 μM	9.75 μM (diethylstilbestrol)
Aqueous solubility (SGF)^a^	>200 μM	—
Partition coefficient (log *D*, *n*-octanol/PBS, pH 7.4)^b^	2.35	1.21 (rifampicin)
Microsomal stability^c^ (half-life/intrinsic clearance)	*t*_1/2_ = 4.5 min Cl_int_ = 1528.7 μL/min/mg protein	*t*_1/2_ = 13.7 min Cl_int_ = 505.7 μL/min/mg protein (terfenadine)
Potassium channel hERG binding	5.2^d^	—

a Tested concentration of 200 μM.

b Tested concentration of 100 μM.

c Tested concentration of 0.1 μM.

d % inhibition at 1 μM.

TC7 showed poor aqueous solubility at physiological pH, whereas its solubility increased significantly in simulated gastric fluid, suggesting a pH-dependent solubility profile. Moreover, the compound displayed a suitable partition coefficient consistent with potential blood–brain barrier (BBB) penetration. However, when permeability was evaluated in MDR1-MDCKII cell monolayers at pH 7.4, TC7 exhibited low bidirectional transport, with apparent permeability coefficients (*P*_app_) below the limit of quantification (*i.e.*, <0.334 × 10^−6^ cm s^−1^ and <0.444 × 10^−6^ cm s^−1^ for the apical-to-basolateral and basolateral-to-apical directions, respectively). This finding was accompanied by very low compound recovery (2% in both transport directions), indicating substantial loss of compound during the assay and narrowing the interpretation of the permeability data (SI, Table S2). Therefore, although the results suggest a limited permeability profile in this *in vitro* model, the low recovery values warrant caution, and additional studies will be required to fully assess the absorption properties of TC7 and its potential interaction with efflux transporters.

Despite its high chemical and enzymatic stability in PBS and DMEM media, TC7 showed very low microsomal stability and high predicted hepatic clearance. The degradation profile obtained in DMEM allowed identification of the ester linker as the most chemically labile moiety of TC7. In particular, the formation of DASA as the major degradation product is consistent with ester bond cleavage, a metabolic pathway commonly observed in human liver microsomes through carboxylesterase-mediated hydrolysis.^30^ Although the DMEM system does not fully replicate hepatic microsomal conditions, these findings provide a mechanistically plausible explanation for the rapid microsomal clearance of TC7; however, additional investigations are needed to fully elucidate the precise metabolic pathway of TC7.

Finally, investigation of potential undesirable cardiovascular side effects for TC7 was assessed through the hERG potassium channel binding. Importantly, no significant affinity was displayed (*i.e.*, % inhibition at 1 μM = 5.2%), indicating a low risk of hERG-related cardiotoxicity.

## Conclusion

In this study, new DASA/HO-1 inhibitor codrugs were designed, synthesized, and preliminarily evaluated *in vitro* for their antiproliferative effect in the U87MG glioblastoma cell line, which expresses high levels of HO-1. Among the tested compounds, TC7 was identified as a promising dual cSRC/HO-1 inhibitor displaying potent inhibitory and antiproliferative activity together with high chemical stability under physiological conditions.

Although the biological evaluation was limited to a single glioblastoma cell line, the findings presented herein provide an initial proof of concept for this codrug strategy and warrant additional experiments in non-tumor astrocytic cells and relevant *in vivo* glioblastoma models to assess its selectivity, efficacy, and translational potential. Nevertheless, the promising results obtained with TC7 identify this compound as a lead candidate for subsequent optimization. Future studies will focus on the development of additional analogues to establish comprehensive structure–activity relationships and further enhance the pharmacological and drug-like properties of this class of compounds. In particular, attention will be devoted to the exploration of alternative cleavable linkers and delivery strategies aimed at improving pharmacokinetic properties and brain targeting.

Overall, the results presented in this work provide a sound basis for the continued preclinical development of this class of compounds as innovative therapeutic candidates for glioblastoma treatment.

## Experimental procedures

### Chemistry

All chemicals and solvents were purchased from commercial vendors and were of reagent grade. DASA was obtained from Fluorochem (product code: F050286). Thin-layer chromatography (TLC) on Merck plates (aluminum sheet coated with silica gel 60 F_254_) was used to monitor the progress of reactions of all the synthesized compounds. Spots were visualized under UV light (*λ* = 254 and 366 nm) or in an iodine chamber. Purification of synthesized compounds by flash column chromatography was performed using Merck silica-gel 60 (230–400 mesh). Melting point measurements were performed in an IA9200 Electrothermal apparatus equipped with a digital thermometer in open glass capillary tubes at a heating rate of 1 °C min^−1^ and were uncorrected. Infrared spectra were determined in KBr disks (solid samples) or NaCl plates (oil samples) on a Perkin Elmer 1600 Series FT-IR spectrometer. The ^1^H NMR and ^13^C NMR spectra of intermediate and final compounds were recorded with a Varian Inova Unity (500 MHz) spectrometer using a DMSO-*d*_6_ solution. The chemical shifts are reported in *δ* values (ppm), using tetramethylsilane (TMS) as the internal standard; the coupling constants (*J*) are given in hertz (Hz). The signal multiplicities are characterized as s (singlet), d (doublet), dd (doublet of doublets), t (triplet), m (multiplet), and br (broad singlet). HRMS spectra were recorded using a Vanquish UHPLC system coupled with an Orbitrap Exploris 120 mass spectrometer (Thermo Scientific). MS full scan acquisitions were performed in positive ion mode, in the *m*/*z* range 150.0–800.0. HCD gas on fragmentation method was used, settled in enhanced resolution, with a maximum injection time of 50 ms. The purity of the final compounds TC7, TC8, and TC9 was determined by RP-HPLC. Analyses were performed with a UHPLC 1260 PRIME Infinity II system (Agilent Technologies, Palo Alto, CA, USA) equipped with a quaternary flexible pump (G7104C), a membrane degasser (G1328C), a diode-array detector (DAD, G7115A), and a fluorescence detector (FLD, G7121B), all integrated into the UHPLC platform. Data acquisition and processing were carried out using OpenLab LC ChemStation software (Agilent Technologies). Separations were achieved on a Kinetex® XB-C18 analytical column (250 × 4.6 mm, 5 μm, 100 Å; Phenomenex) maintained at room temperature. The mobile phase consisted of acetonitrile containing 0.1% trifluoroacetic acid (solvent A) and water containing 0.1% trifluoroacetic acid (solvent B). The injection volume was 20 μL, and the flow rate was 1.0 mL min^−1^ under the following gradient elution conditions. For TC7 (*t*_R_ = 20.7 min): 10% A for 5 min; 10–20% A from 5 to 8 min; 20% A until 9 min; 20–40% A from 9 to 18 min; and 40% A until 24 min, followed by a 2 min re-equilibration between runs. For TC8 (*t*_R_ = 30.7 min) and TC9 (*t*_R_ = 22.7 min): 10% A for 5 min; 10–20% A from 5 to 8 min; 20% A until 9 min; 20–40% A from 9 to 18 min; and 40% A until 30 min, followed by a 2 min re-equilibration between runs. All compounds were dissolved in the mobile phase at a concentration of about 0.01 mg mL^−1^. The column effluent was monitored using the DAD. The DAD acquired the UV spectra in the range from 190 to 800 nm, and the UHPLC chromatogram was recorded at 230, 254, and 325 (with 800 nm as the reference wavelength). The purity of the test samples was evaluated as the percentage ratio between the areas of the main peak and of possible impurities at the three wavelengths and also using a DAD purity analysis of the chromatographic peak. The purity of all the target compounds was found to be ≥95%.

### 4-(2-(4-(6-((5-((2-Chloro-6-methylphenyl)carbamoyl)thiazol-2-yl)amino)-2-methylpyrimidin-4-yl)piperazin-1-yl)ethoxy)-4-oxobutanoic acid (DASA-succ)

DASA (100 mg, 0.223 mmol) was dissolved in anhydrous DMF (2 mL) in a 25 mL round-bottom flask sealed with a rubber septum and flushed with nitrogen. To the stirred solution thus obtained, succinic anhydride (22.3 mg, 0.223 mmol) and TEA (343 μL, 0.247 mmol) were added. The reaction was carried out for 24 h at 60 °C. The mixture was quenched with water (30 mL) and extracted with ethyl acetate (3 × 10 mL). The collected organic layer was dried over anhydrous Na_2_SO_4_ and evaporated to dryness under reduced pressure to obtain DASA-succ (61.3 mg, 52%) as a white pure solid. Mp: 242.6–244.5 °C; IR (KBr, selected lines): *ν̃* (cm^−1^) = 3191 (N–H), 2958, 1738 (C=O), 1615, 1574, 1505, 1413, 1295 (C–O), 1195, 984, 860; ^1^H NMR (500 MHz, DMSO-*d*_6_): *δ* 11.45 (br, 1H, NHCO), 9.85 (s, 1H, NH), 8.20 (s, 1H, Ar), 7.38 (d, *J* = 7.6 Hz, 1H, Ar), 7.31–7.19 (m, 2H, Ar), 6.04 (s, 1H, Ar), 4.14 (t, *J* = 5.8 Hz, 2H, CH_2_OCO), 3.49 (br, 2H, piperazine), 2.57 (t, *J* = 5.8 Hz, 2H, N*CH*_2_CH_2_OCO), 2.51–2.44 (m, 10H, piperazine + succinic), 2.39 (s, 3H, CH_3_), 2.22 (s, 3H, ArCH_3_); ^13^C NMR (126 MHz, DMSO-*d*_6_): *δ* 173.4, 172.1, 165.1, 162.5, 162.3, 159.9, 156.9, 140.8, 138.8, 133.5, 132.4, 129.0, 128.1, 127.0, 125.7, 82.6, 61.5, 55.9, 52.4, 43.6, 28.8, 28.8, 25.6, 18.3. HRMS (ESI) *m*/*z*: [M + H]^+^ calcd for C_26_H_30_ClN_7_O_5_S 588.1790; found 588.1781.

### General procedure for the synthesis of codrugs TC7, TC8, and TC9

To a stirred solution of DASA-succ (1.2 equiv.) in a mixture of dry DMF/DCM (3 and 1 mL, respectively), the appropriate alcohol derivative SI 1/09, LS 6/42, or LS 4/28 (1.0 equiv.), EDC·HCl (1.2 equiv.), and DMAP (0.1 equiv.) were added. The obtained reaction mixture was stirred at room temperature for 24 h under a nitrogen flow. Afterward, the mixture was diluted with 10% aqueous NaCl solution (75 mL) and the aqueous phase was extracted with ethyl acetate (25 mL). The organic layer was dried with anhydrous Na_2_SO_4_ and concentrated under reduced pressure. The resulting crude product was then purified by flash column chromatography on silica gel with a mixture of ethyl acetate/methanol (7 : 3, v/v) as eluent. The residue was triturated with petroleum ether to give the final compounds as pure solids. The following compounds were obtained using this procedure:

#### 1-(3-Bromophenyl)-2-(1*H*-imidazol-1-yl)ethyl (2-(4-(6-((5-((2-chloro-6-methylphenyl)carbamoyl)thiazol-2-yl)amino)-2-methylpyrimidin-4-yl)piperazin-1-yl)ethyl) succinate (TC7)

The title compound was obtained as a white solid (102 mg, 33%); mp: 180.8–181.8 °C; IR (KBr, selected lines): *ν̃* (cm^−1^) = 3432 (N–H), 3193 (N–H), 2944, 1740 (C=O), 1619 (C=N), 1578, 1507, 1414, 1292 (C–O), 1209 (C–O), 1006, 813; ^1^H NMR (500 MHz, DMSO-*d*_6_): *δ* 11.46 (s, 1H, NHCO), 9.87 (s, 1H, NH), 8.22 (s, 1H, Ar), 7.55 (s, 1H, imidazole), 7.54–7.50 (m, 2H, Ar), 7.40 (d, *J* = 7.7, 1H, Ar), 7.35–7.31 (m, 2H, Ar), 7.31–7.22 (m, 2H, Ar), 7.11 (s, 1H, imidazole), 6.85 (s, 1H, imidazole), 6.05 (s, 1H, Ar), 5.97 (t, *J* = 6.6, 1H, *CH*OCH_2_), 4.42–4.34 (m, 2H, CHO*CH*_2_), 4.13 (t, *J* = 5.8 Hz, 2H, CH_2_OCO), 3.49 (br, 4H, piperazine), 2.68–2.51 (m, 6H, N*CH*_2_CH_2_OCO + succinic), 2.49–2.44 (m, 4H, piperazine), 2.41 (s, 3H, CH_3_), 2.24 (s, 3H, ArCH_3_); ^13^C NMR (126 MHz, DMSO-*d*_6_): *δ* 171.7, 170.8, 165.2, 162.6, 162.3, 159.9, 156.9, 140.8, 139.8, 138.8, 137.8, 133.5, 132.4, 131.2, 130.6, 129.1, 129.0, 128.2, 128.16, 127.0, 125.7, 125.3, 121.7, 120.0, 82.6, 73.7, 61.6, 55.9, 52.3, 50.2, 43.5, 28.8, 28.5, 25.6, 18.3. HRMS (ESI) *m*/*z*: [M + H]^+^ calcd for C_37_H_39_BrClN_9_O_5_S 836.1740; found 836.1727.

#### 1-(4-((4-Bromobenzyl)oxy)phenyl)-2-(1*H*-imidazol-1-yl)ethyl (2-(4-(6-((5-((2-chloro-6-methylphenyl)carbamoyl)thiazol-2-yl)amino)-2-methylpyrimidin-4-yl)piperazin-1-yl)ethyl) succinate (TC8)

The title compound was obtained as a white solid (103 mg, 36%); mp: 178.3–174.4 °C; IR (KBr, selected lines): *ν̃* (cm^−1^) = 3235 (N–H), 3021, 2944, 1734 (C=O), 1611, 1578, 1508 (C=C), 1456, 1431, 1281 (C–O), 1195 (C–O), 1155, 1008, 807; ^1^H NMR (500 MHz, DMSO-*d*_6_): *δ* 11.48 (s, 1H, NHCO), 9.90 (s, 1H, NH), 8.24 (s, 1H, Ar), 7.57 (d, *J* = 8.1 Hz, 2H, Ar), 7.52 (s, 1H, imidazole), 7.43–7.34 (m, 3H, Ar), 7.34–7.19 (m, 4H, Ar), 7.11 (s, 1H, imidazole), 6.98 (d, *J* = 8.4 Hz, 2H, Ar), 6.84 (s, 1H, imidazole), 6.06 (s, 1H, Ar), 5.92 (dd, *J* = 7.6, 4.4 Hz, 1H, *CH*OCH_2_), 5.07 (s, 2H, CH_2_O), 4.41–4.27 (m, 2H, CHO*CH*_2_), 4.11 (t, *J* = 5.9 Hz, 2H, CH_2_OCO), 3.48 (br, 4H, piperazine), 2.61–2.48 (m, 6H, N*CH*_2_CH_2_OCO + succinic), 2.48–2.43 (m, 4H, piperazine), 2.41 (s, 3H, CH_3_), 2.24 (s, 3H, ArCH_3_); ^13^C NMR (126 MHz, DMSO-*d*_6_): *δ* 171.7, 170.8, 165.2, 162.6, 162.4, 159.9, 158.1, 156.9, 140.8, 138.8, 137.8, 136.4, 133.5, 132.4, 131.3, 129.7, 129.5, 129.0, 128.2, 127.8, 127.0, 125.7, 120.9, 119.9, 114.7, 109.5, 82.6, 74.2, 68.4, 61.6, 55.8, 52.3, 50.3, 43.6, 28.8, 28.6, 25.6, 18.3. HRMS (ESI) *m*/*z*: [M + H]^+^ calcd for C_44_H_45_BrClN_9_O_6_S 942.2158; found 942.2138.

#### 1-([1,1′-Biphenyl]-3-yl)-2-(1*H*-imidazol-1-yl)ethyl (2-(4-(6-((5-((2-chloro-6-methylphenyl)carbamoyl)thiazol-2-yl)amino)-2-methylpyrimidin-4-yl)piperazin-1-yl)ethyl) succinate (TC9)

The title compound was obtained as a white solid (88.1 mg, 20%); mp: 172.3–173.5 °C; IR (KBr, selected lines): *ν̃* (cm^−1^) = 3206 (N–H), 3094, 3045, 1734 (C=O), 1618 (C=N), 1578, 1506 (C=C), 1414, 1292 (C–O), 1194 (C–O), 1155, 1005, 985, 757; ^1^H NMR (500 MHz, DMSO-*d*_6_): *δ* 11.47 (s, 1H, NHCO), 9.88 (s, 1H, NH), 8.22 (s, 1H, Ar), 7.68–7.63 (m, 2H, Ar), 7.63–7.57 (m, 2H, Ar), 7.54 (s, 1H, imidazole), 7.50–7.42 (m, 3H, Ar), 7.42–7.35 (m, 2H, Ar), 7.33 (d, *J* = 7.7 Hz, 1H, Ar), 7.31–7.21 (m, 2H, Ar), 7.15 (s, 1H, imidazole), 6.87 (s, 1H, imidazole), 6.09–5.98 (m, 2H, Ar + *CH*OCH_2_), 4.49–4.38 (m, 2H, CHO*CH*_2_), 4.09 (t, *J* = 5.9 Hz, 2H, CH_2_OCO), 3.46 (br, 4H, piperazine), 2.69–2.54 (m, 4H, succinic), 2.46–2.41 (m, 6H, N*CH*_2_CH_2_OCO + piperazine), 2.40 (s, 3H, CH_3_), 2.24 (s, 3H, ArCH_3_); ^13^C NMR (126 MHz, DMSO-*d*_6_): *δ* 171.8, 170.9, 165.1, 162.6, 162.3, 159.9, 156.9, 140.4, 139.8, 138.8, 137.9, 133.5, 132.4, 129.04, 129.01, 128.9, 128.2, 127.6, 127.0, 126.8, 126.6, 125.7, 125.2, 124.6, 120.0, 109.5, 82.6, 74.5, 61.5, 55.8, 52.3, 50.5, 43.5, 28.8, 28.6, 25.6, 18.3. Anal. calcd for C_43_H_44_ClN_9_O_5_S: C, 61.90; H, 5.32; N, 15.11. Found: C, 61.78; H, 5.30; N, 15.07. HRMS (ESI) *m*/*z*: [M + H]^+^ calcd for C_43_H_44_ClN_9_O_5_S 834.2947; found 834.2935.

### Molecular modelling studies

Molecular docking was performed using the FRED (Fast Rigid Exhaustive Docking) module as implemented within the OpenEye software (Cadence Molecular Sciences, Inc., Santa Fe, NM). This docking tool depends on a multi-conformer algorithm to generate a set of low-energy conformers submitted for rigid docking.^31^ The input includes a file of the prepared receptor and a database of the generated conformers. The 2.19 Å crystal structure of HO-1 (PDB ID 3HOK) as well as the 2.20 Å crystal structure of the kinase domain of cSRC in complex with DASA (PDB ID 3G5D) was used for the docking simulation. The make-receptor command was used in FRED to prepare the receptor grid for docking, while the conformer library of the prepared compounds TC7 to TC9 was generated using the default settings of the Omega module, also available within OpenEye. The docking grid was centered on the sidechain carbonyl of Asp140 and the backbone carbonyl of Met341 in HO-1 and cSRC, respectively. FRED software employs the Chemgauss4 scoring function, which depends on shape complementarity. The score is composed of the following energy terms: steric, acceptor, donors, coordinating groups, metals, lone pairs, polar hydrogens, and chelator coordinating groups. Visual examination and selection of the best poses were performed using OpenEye VIDA 2023.2.3 and PyMOL 2.5.5 graphical software modules.

### Biological evaluation

#### Cell cultures and viability assay

Experiments were conducted on human GBM cell line U87MG (ATCC number HTB-14) (purchased from ATCC, Rockville, MD, USA). Cells were cultured as previously described.^32^ To evaluate the effect of parent drugs and final compounds on cell viability, GMB cells were seeded into 96-well plates at a density of 7.0 × 10^3^ cells per well in 100 μL of culture medium. Subsequently, cells were treated for 48 h with the tested compounds at different concentrations (0.1–10 μM). Following treatments, 0.5 mg mL^−1^ of 3-[4,5-dimethylthiazol-2-yl]-2,5-diphenyltetrazolium bromide (MTT) (Sigma-Aldrich) was added to each well and incubated for 3 h at 37 °C. Finally, dimethyl sulfoxide (DMSO) was used to dissolve formazan salts, and absorbance was measured at 570 nm in a microplate reader (Biotek Synergy-HT, Winooski, VT, USA). Eight replicate wells were used for each group, and at least three separate experiments were performed.

#### Preparation of spleen microsomal fractions

Rat spleen microsomal fractions were prepared by differential centrifugation to obtain the HO-1 enzyme. Rat spleen microsomal fractions were used to reflect the activity of most native (*i.e.*, closest to *in vivo*) forms of HO-1. Spleen (Sprague-Dawley rats) microsomal fractions were prepared according to the procedure outlined by Ryter *et al.*^33^ The experiments reported in the present paper complied with current Italian law and met the guidelines of the Institutional Animal Care and Use Committee of the Ministry of Health (Directorate General for Animal Health and Veterinary Medicines) (Italy). The experiments were performed in male Sprague-Dawley albino rats (150 g body weight and age 45 days). Animals had free access to water and were maintained in a temperature- and light-controlled facility. After animals were sacrificed, their spleen was excised, weighed, and pooled to obtain homogenates. Spleen homogenates (15%, w/v) pooled from five rats were prepared in ice-cold buffer (50 mM Tris buffer, pH 7.4, containing 0.25 M sucrose) using a Potter-Elvehjem homogenizing system with a Teflon pestle. Centrifugation at 10 000*g* for 20 min at 4 °C, followed by centrifugation of the supernatant at 100 000g for 60 min at 4 °C, was used to obtain microsomal fractions of rat spleen homogenates. The 100 000*g* pellet (microsomes) was resuspended in 100 mM potassium phosphate buffer, pH 7.8, containing 2 mM MgCl_2_ with a Potter-Elvehjem homogenizing system. Equal aliquots of rat spleen microsomal fractions were aliquoted and stored at −80 °C for up to 2 months. Protein concentration was measured using TAKE 3 nanodrop plate.

#### Preparation of biliverdin reductase (BVR)

Liver cytosol was used as a source of BVR. Rat liver was perfused through the hepatic portal vein with cold 0.9% NaCl, then it was cut and flushed with ice-cold PBS to remove all the blood. Liver tissue was homogenized in 3 volumes of a solution containing 1.15% KCl (w/v) and Tris buffer 20 mM, pH 7.8 on ice. Homogenates were centrifuged at 10 000*g* for 20 min at 4 °C. The supernatant was decanted and centrifuged at 100 000*g* for 1 h at 4 °C to sediment the microsomes. The 100 000*g* supernatant was saved and then stored in aliquots at −80 °C after protein concentration measurement. The prepared BVR was subsequently used in the HO activity assay to catalyse the conversion of biliverdin to bilirubin, enabling the spectrophotometric quantification of HO activity.

#### Measurement of HO enzymatic activities in the microsomal fraction of rat spleen and in cells

HO enzymatic activity was tested both in a cell-free model and in U87MG cell lysates. As sources of HO-1 spleen microsomal fractions were used and enzymatic activity was assessed as previously reported.^20^ U87MG cells were treated with the tested compounds at a concentration of 5 μM and harvested after 48 h; protein levels in cell lysates were quantified to evaluate HO enzymatic activity by measuring the bilirubin formation through the difference in absorbance at 464 to 530 nm. Reaction mixtures consisted of 20 mM Tris-HCl (pH 7.4), cell lysate (2 mg mL^−1^), 0.5–2 mg mL^−1^ biliverdin reductase, 1 mM NADPH, 2 mM glucose 6-phosphate (G6P), 1 U G6P dehydrogenase and 25 μM hemin. Incubation was carried out in a circulating water bath in the dark for 1 h at 37 °C. The reaction was stopped by adding chloroform (1 : 1). After chloroform phase recovery, the amount of formed bilirubin was measured with a double-beam spectrophotometer at OD 464–530 nm (extinction coefficient, 40 mM cm^−1^ for bilirubin). One unit of the enzyme was defined as the amount of enzyme catalysing the formation of 1 nmol of bilirubin per mg protein per h.

#### Measurement of ROS levels

Levels of ROS were determined using the fluorescent probe 2′,7′-dichlorofluorescein diacetate (DCFH-DA). Following 3 h of treatment with TC7, SI 1/09 and DASA, cells were washed with a 0.1% Triton solution to enhance cellular probe permeation, then 100 μL of a DCFH-DA working solution (200 μM) was added to each well and incubated at 37 °C for 0.5 h. After incubation, fluorescence was measured in a microplate reader (excitation, *λ* = 488 nm; emission, *λ* = 525 nm). Eight replicate wells were used for each group. The results are expressed as fluorescence intensity/cell number.

#### Measurement of HO-1 protein levels

HO-1 levels were assessed after 24 h of treatment on U87MG cell lysates using enzyme-linked immunosorbent assays (ELISA) (ab207621, Abcam, Cambridge, UK) according to the manufacturer's instructions. The absorbance was measured using a microplate reader (Biotek Synergy-HT, Winooski, VT, USA). The results are expressed as pg mL^−1^ per protein.

#### Determination of thiol groups

The concentration of non-protein thiol groups (RSH), reflecting about 90% of the GSH cellular content, was measured in U87MG total cell lysates. After 24 h of treatment, RSH levels were evaluated by a spectrophotometric assay based on the reaction of thiol groups with 2,2-dithio-*bis*-nitrobenzoic acid (DTNB). A DTNB solution and the samples were mixed and incubated at room temperature for 20 min in the dark until the noticeable appearance of a yellow color. After incubation, samples were centrifuged at 3000 rpm for 10 min. The supernatant was collected and set in a black 96-well plate for measurement of the absorbance in a microplate reader (Biotek Synergy-HT, Winooski, VT, USA) at *λ* = 412 nm. Experiments were conducted in quadruplicate. The results are expressed in pmoles per μL per protein.

#### Statistical analysis

At least three independent experiments were performed for each analysis. A one-way analysis of variance (ANOVA) was used to compare differences among groups, followed by the Tukey–Kramer *post hoc* test. Data are presented as the mean ± standard error (SEM). The level of significance for all the statistical tests was set at *p* < 0.05.

All animal procedures were performed in accordance with the Guidelines for Care and Use of Laboratory Animals and were approved by the OPBA (Organismo Preposto al Benessere degli Animali) of the University of Catania.

### Chemical and enzymatic stability

#### Stability in PBS

The chemical stability of TC7 was evaluated *in vitro* following an incubation protocol based on previously published methods.^13,34^TC7 was first dissolved in DMSO and then diluted with phosphate-buffered saline (50 mM, pH 7.4) in glass vials to a final concentration of 25 μM. All experiments were performed in triplicate. Samples were incubated at 37 ± 0.5 °C for 24 h, and 20 μL aliquots were collected at selected time points for analysis by HPLC, as described below. Experiments were conducted on a 5 mL scale, without replacement of the collected aliquots. Data are reported as the concentrations (μM) of unmodified TC7 and the released parent compound DASA over time.

#### Stability in DMEM

The enzymatic stability of TC7 was assessed in DMEM supplemented with fetal bovine serum using an *in vitro* incubation assay adapted from our previously reported methodology.^13,35^ A stock solution of TC7 in DMSO was diluted in pre-warmed DMEM (37 °C) to a final concentration of 25 μM. Samples were incubated at 37 ± 0.5 °C for 26 h, and at selected time points, 300 μL aliquots were collected and immediately mixed with an equal volume of acetonitrile containing 0.1% trifluoroacetic acid to precipitate proteins. After sonication and vigorous mixing, the samples were centrifuged at 2150*g* for 10 min at 4 °C. The resulting supernatants were filtered through 0.45 μm PTFE membranes and analyzed by HPLC as described below. Data are reported as concentrations (μM) of unmodified TC7 and the released parent compound DASA over time.

#### HPLC analysis

The reverse-phase HPLC method enabled the effective separation and quantification of TC7 and its parent compound DASA. The chromatographic conditions are reported above. The column effluent was monitored at 230, 254, and 325 nm with reference at 800 nm. Quantitative analysis was performed at 325 nm (reference wavelength 800 nm), corresponding to the maximum absorbance of both TC7 and DASA, ensuring optimal sensitivity and selectivity. Calibration curves were generated using external standards, showing good linearity over the concentration range of 0.93–29.89 μM for TC7 and 1.60–51.23 μM for DASA (*r*^2^ >0.99).

#### *In vitro* ADMET assays

*In vitro* ADMET assays were performed at Eurofins Discovery (https://www.eurofins.com/) according to their experimental protocols for the corresponding items: aqueous solubility in PBS (item number 435), aqueous solubility in simulated gastric fluid (item number 2061), partition coefficient (item number 417), intrinsic clearance in liver microsome-human (item number 607), hERG human potassium ion channel binding (catalogue product number 4094), and bidirectional permeability MDR1-MDCKII (item number G363).

## Author contributions

S. I.: conceptualization. S. I., A. G. D., V. S., F. S., and M. A. H.: participated in research design. L. S., V. P., M. N. M., G. R., S. M., C. T. and S. I.: synthesized, purified, and characterized all compounds. V. P., L. V., and S. I.: conducted formal analysis. A. G. D.: performed *in vitro* antiproliferative tests. V. C., G. C., and L. V.: conducted *in vitro* biochemistry assays. M. A. H.: performed molecular modeling studies. M. E. S. and F. S.: performed *in vitro* chemical and metabolic stability studies. V. S., and S. I.: contributed reagents, materials, and analysis tools. A. G. D., V. C., F. S., and S. I.: performed data analysis. S. I. and G. R.: acquired the funding. S. I.: writing of original draft. L. S., V. C., F. S., M. A. H., and S. I.: wrote, reviewed and edited the manuscript. All authors reviewed and approved the final version of the manuscript.

## Conflicts of interest

The authors declare no competing financial interest.

## Supplementary Material

MD-OLF-D6MD00227G-s001

## Data Availability

The primary data supporting this work has been included as part of the supplementary information (SI). Supplementary information: ^1^H NMR spectra, APT spectra, HRMS, and HPLC spectra of the compounds. See DOI: https://doi.org/10.1039/d6md00227g.
