# Supplementary material for: Design, synthesis, and in vitro antiproliferative evaluation of dasatinib/HO-1 inhibitor codrugs towards glioblastoma therapy
Source: RSC Med Chem. 2026 Oct 7. Online ahead of print. doi: 10.1039/d6md00227g (PMC13684878; doi:10.1039/d6md00227g)
Supplement: Supplementary file 1 [file MD-OLF-D6MD00227G-s001.pdf]

Supplementary Information (SI) for RSC Medicinal Chemistry.
This journal is © The Royal Society of Chemistry

Supporting Information

Design, Synthesis, and In Vitro Antiproliferative Evaluation of Dasatinib/HO-1 Inhibitor Codrugs Towards Glioblastoma Therapy

Valeria Pittalà [a], Loredana Salerno [a], Valeria Consoli [a], Agata Grazia D'Amico [a], Valeria Sorrenti [a], Luca Vanella [a], Giorgia Centamore [a], Chiara Terlati [a], Sara Mocka [a], Maria Nunziata Modica [a], Giuseppe Romeo [a], Marica Erminia Schiano [b], Federica Sodano [b], Mohamed A. Helal [c, d], Sebastiano Intagliata [a*]

[a] Department of Drug and Health Sciences, University of Catania, viale A. Doria 6, 95125, Catania, Italy.

[b] Department of Pharmacy, "Federico II" University of Naples, 80131 Naples, Italy.

[c] Biomedical Sciences Program, University of Science and Technology, Zewail City of Science and Technology, Giza 12587, Egypt.

[d] Medicinal Chemistry Department, Faculty of Pharmacy, Suez Canal University, Ismailia 41522, Egypt.

*Corresponding author: s.intagliata@unict.it; Tel.: +39-095-738-4053

Table of contents:

^{1}H NMR, APT, and HRMS spectra of DASA-succ and final compounds TC7, TC8, TC9 (**Figures S1–S2**)	p. S2–S7
Docking scores for selected docked poses (**Table S1**)	p. S8
Effect of DASA, SI 1/09, combo, and TC7 (5 μM) on A172 cells viability at 48h (**Figure S13**)	p. S8
HPLC-chromatogram of TC7 after 24h of incubation in PBS and DMEM, respectively (**Figure S14** and **S15**)	p. S9
In vitro absorption (**Table S2**)	p. S9

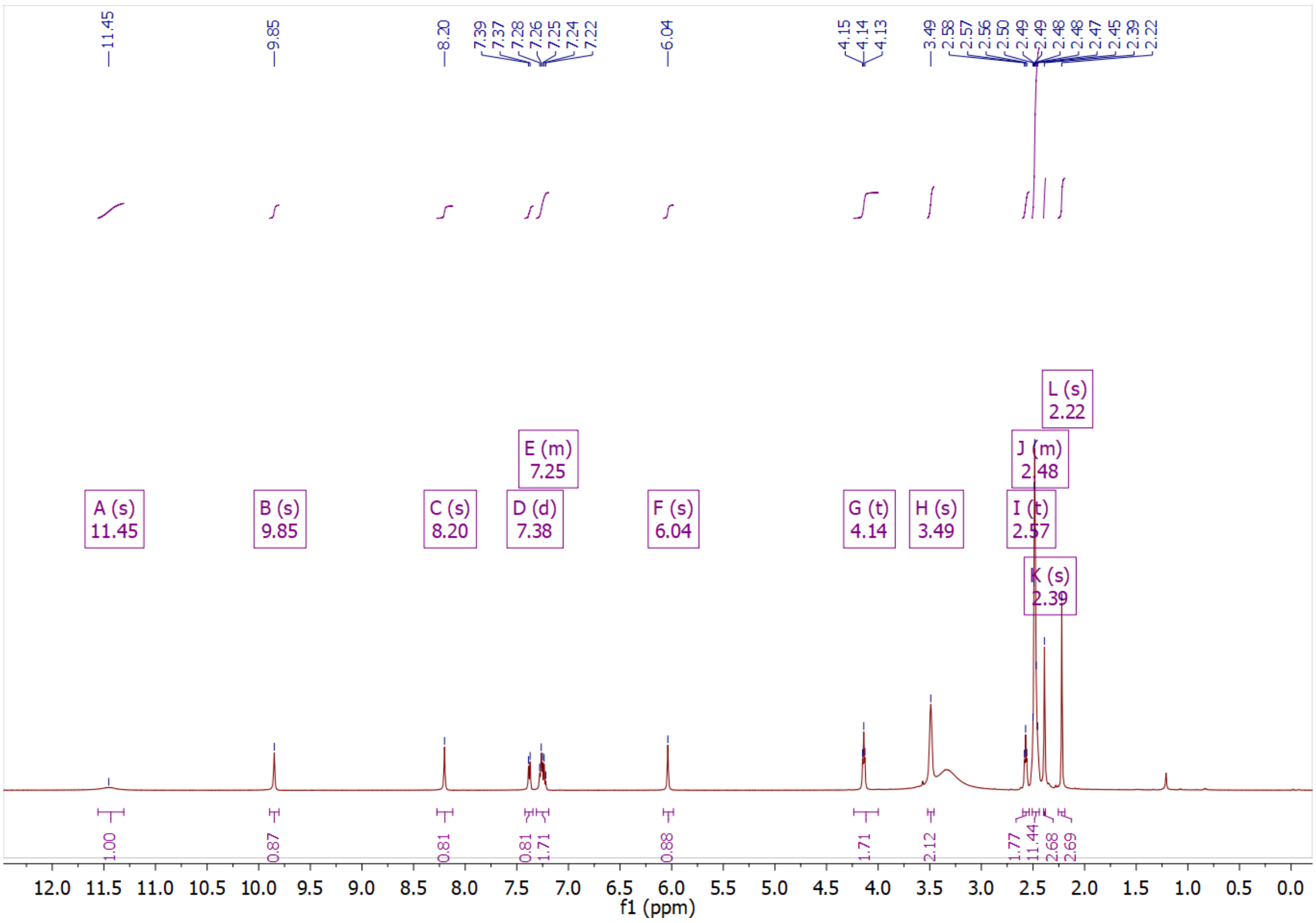

Figure S1. ^{1}H NMR (500 MHz, DMSO-d_6) spectrum of DASA-succ.

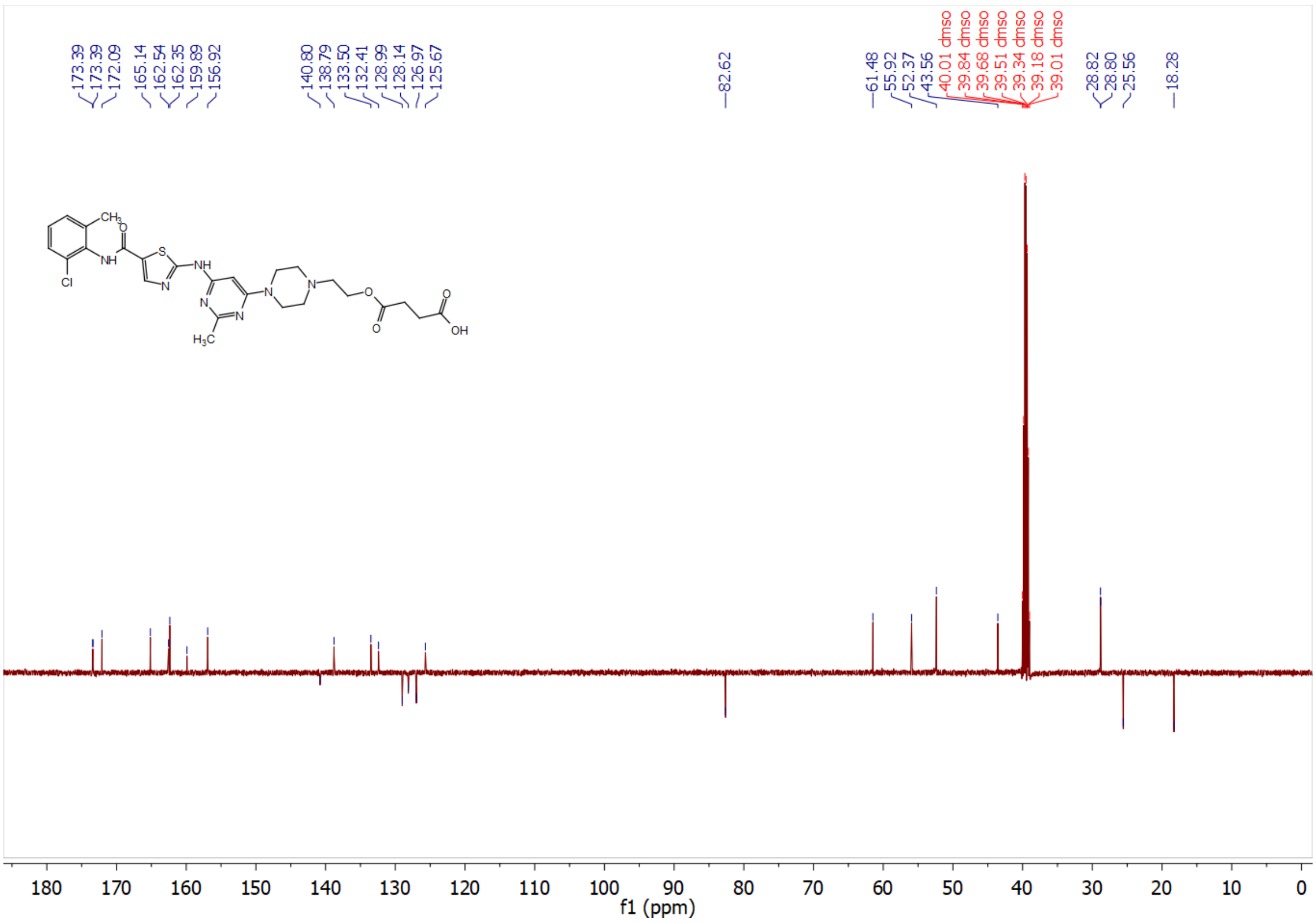

Figure S2. APT (126 MHz, DMSO-d_6) spectrum of DASA-succ.

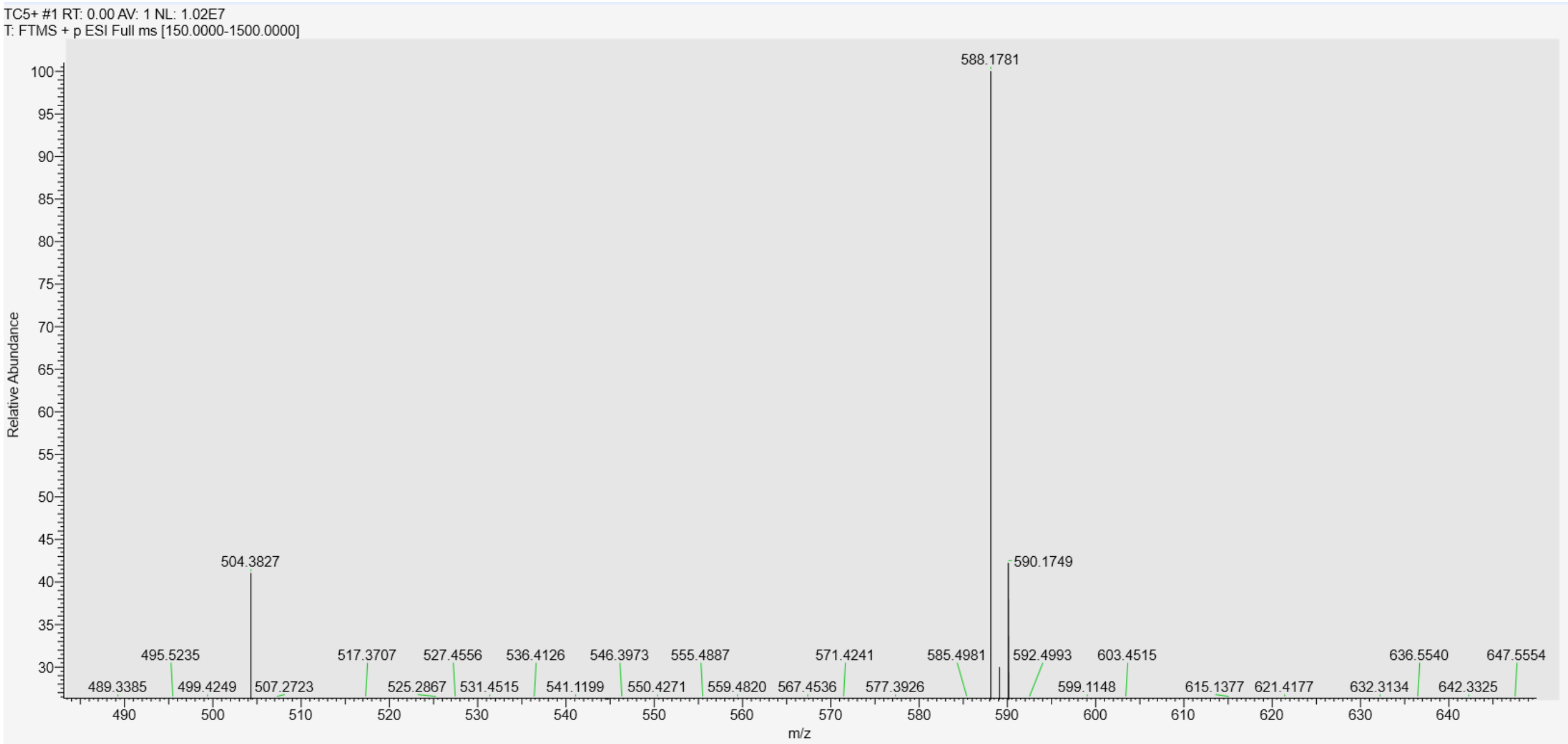

Figure S3. HRMS spectrum of DASA-succ.

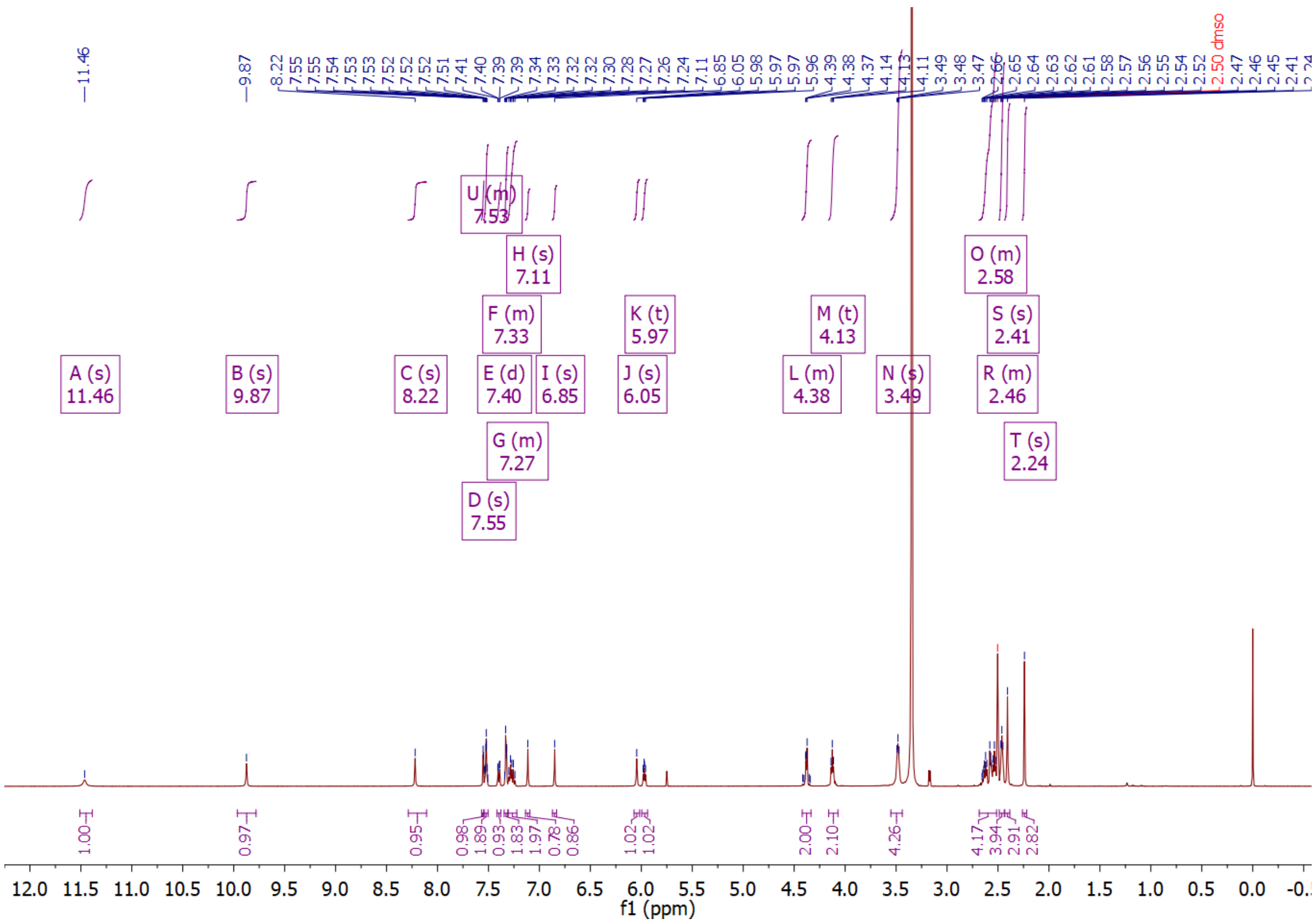

Figure S4. ^{1}H NMR (500 MHz, DMSO-d_6) spectrum of TC7.

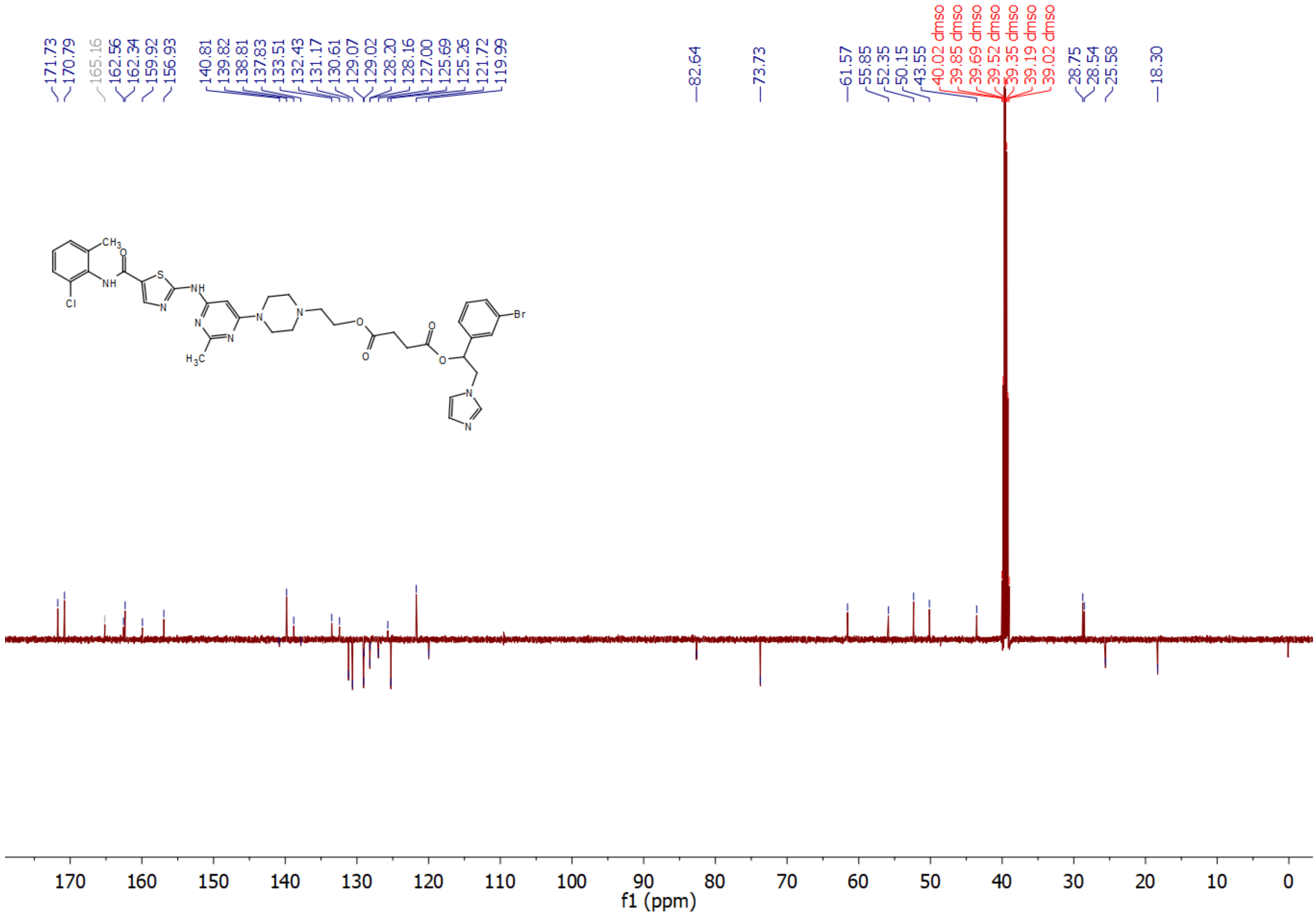

Figure S5. APT (126 MHz, DMSO-d_6) spectrum of TC7.

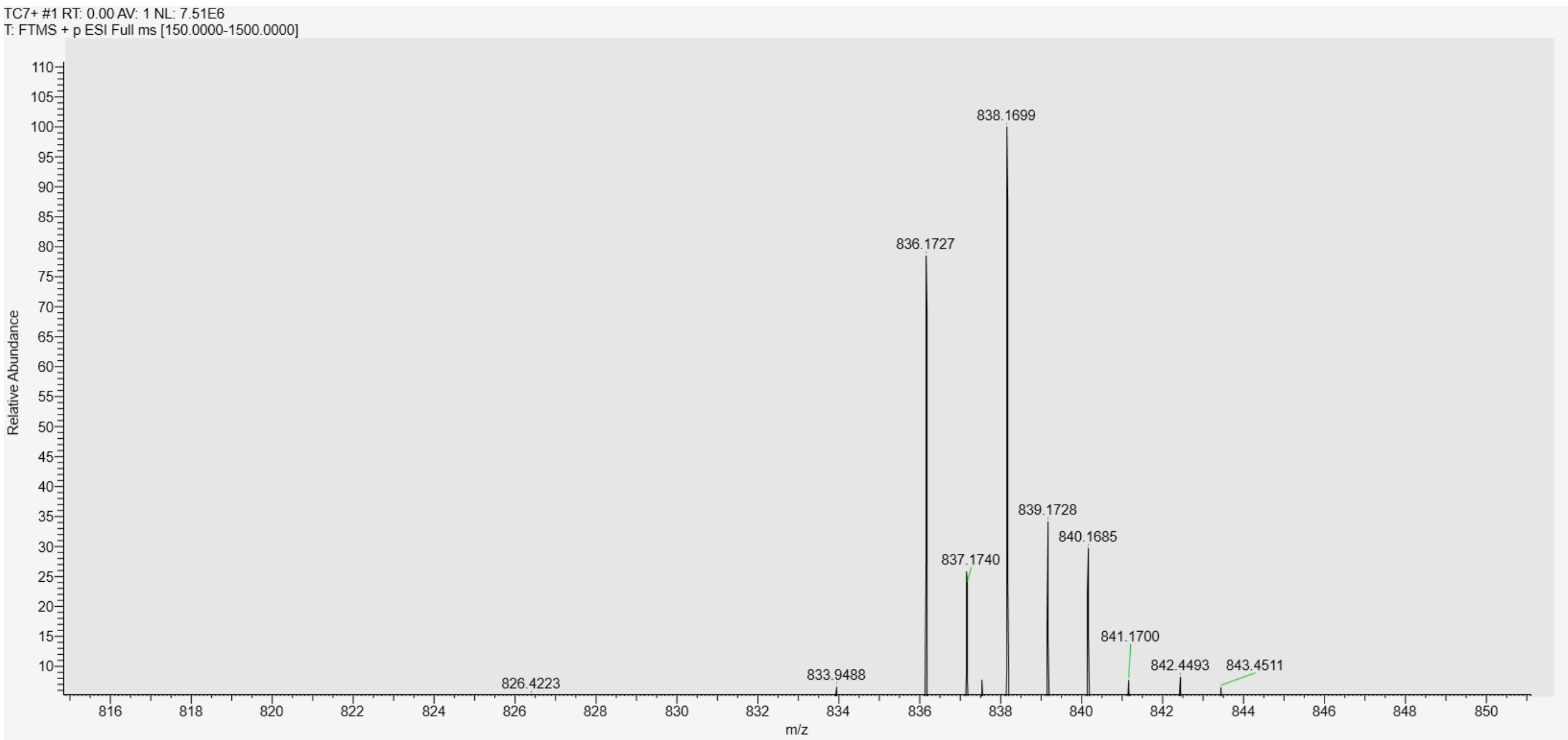

Figure S6. HRMS spectrum of TC7.

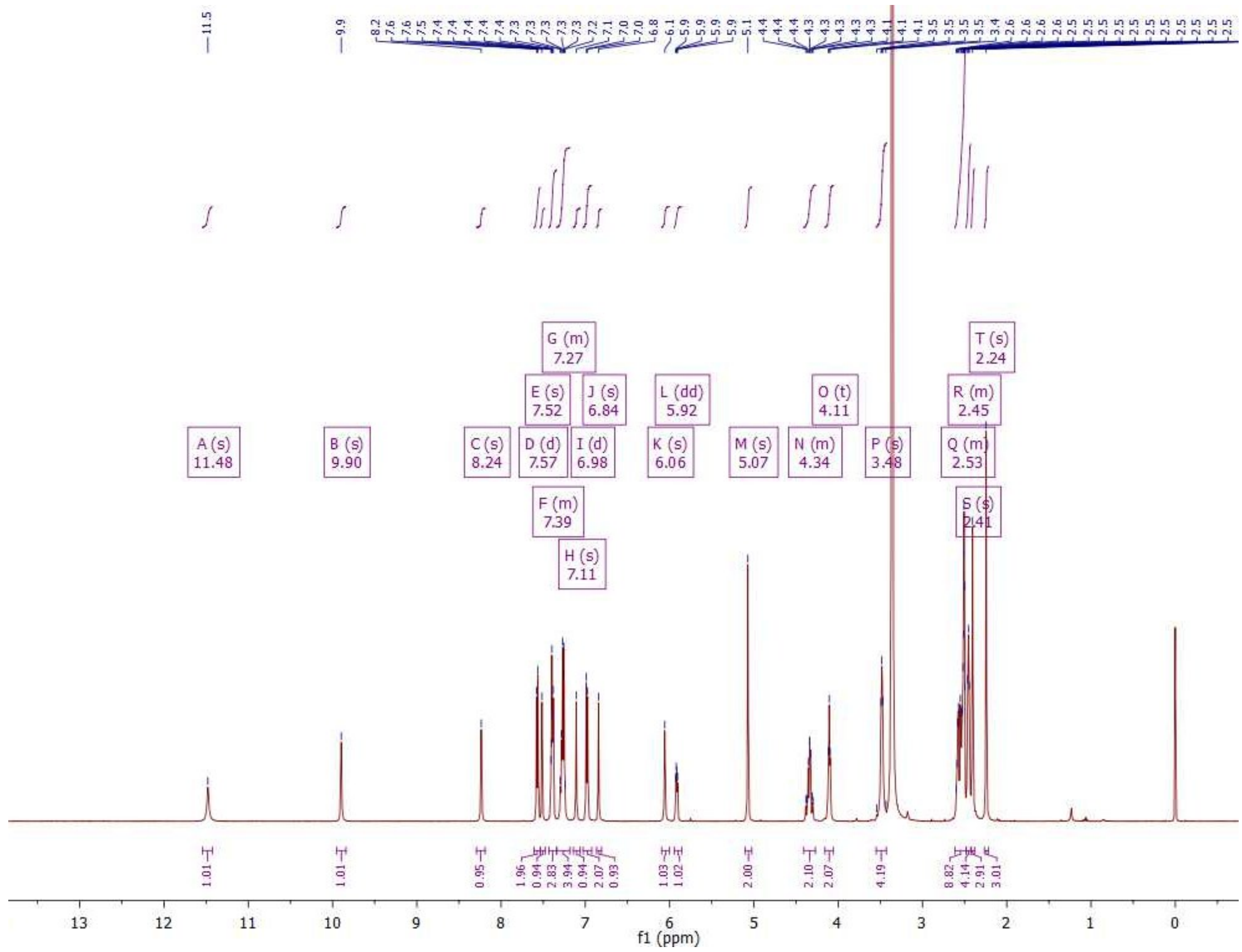

Figure S7. ^{1}H NMR (500 MHz, DMSO-d_6) spectrum of TC8.

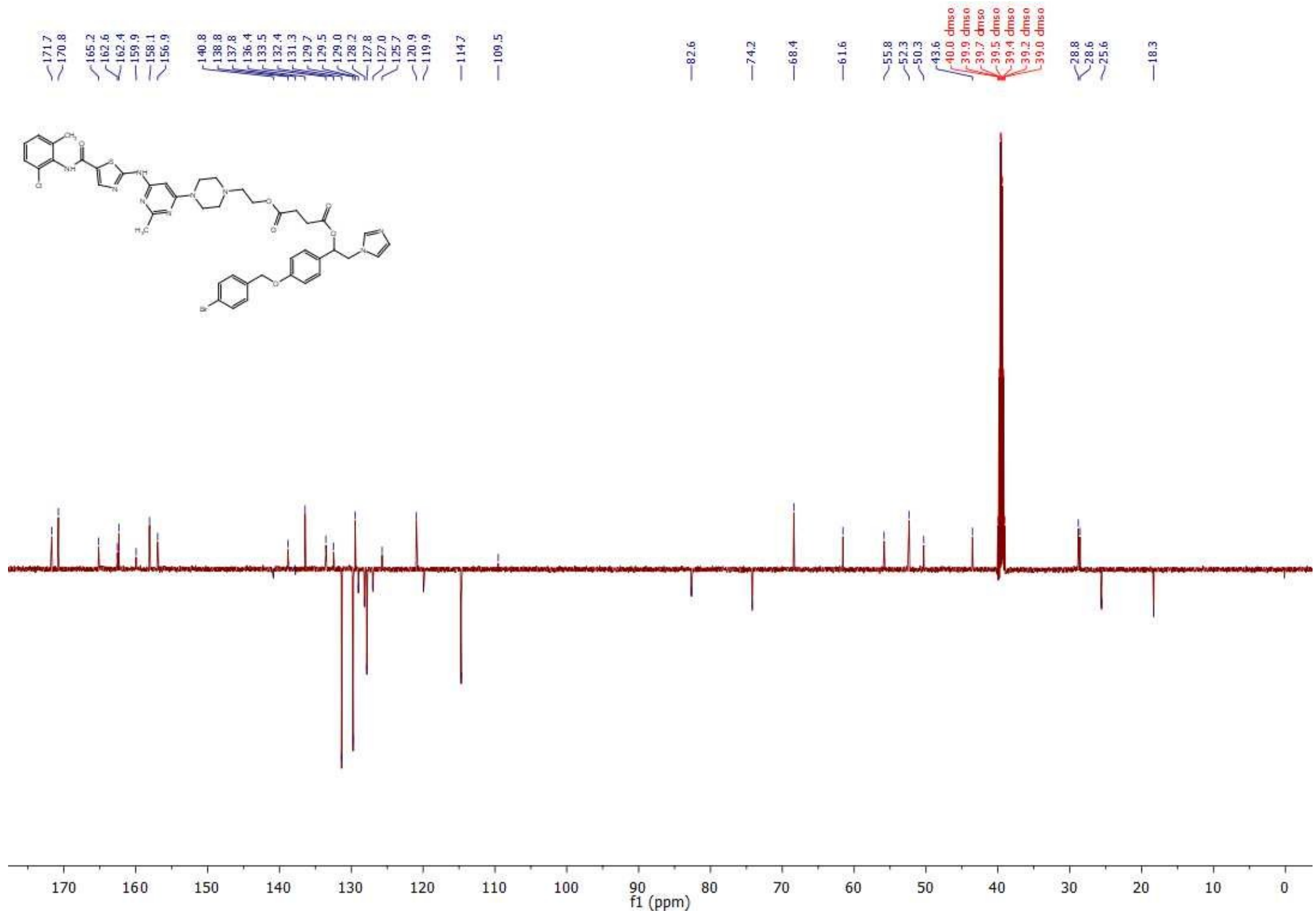

Figure S8. APT (126 MHz, DMSO-d_6) spectrum of TC8.

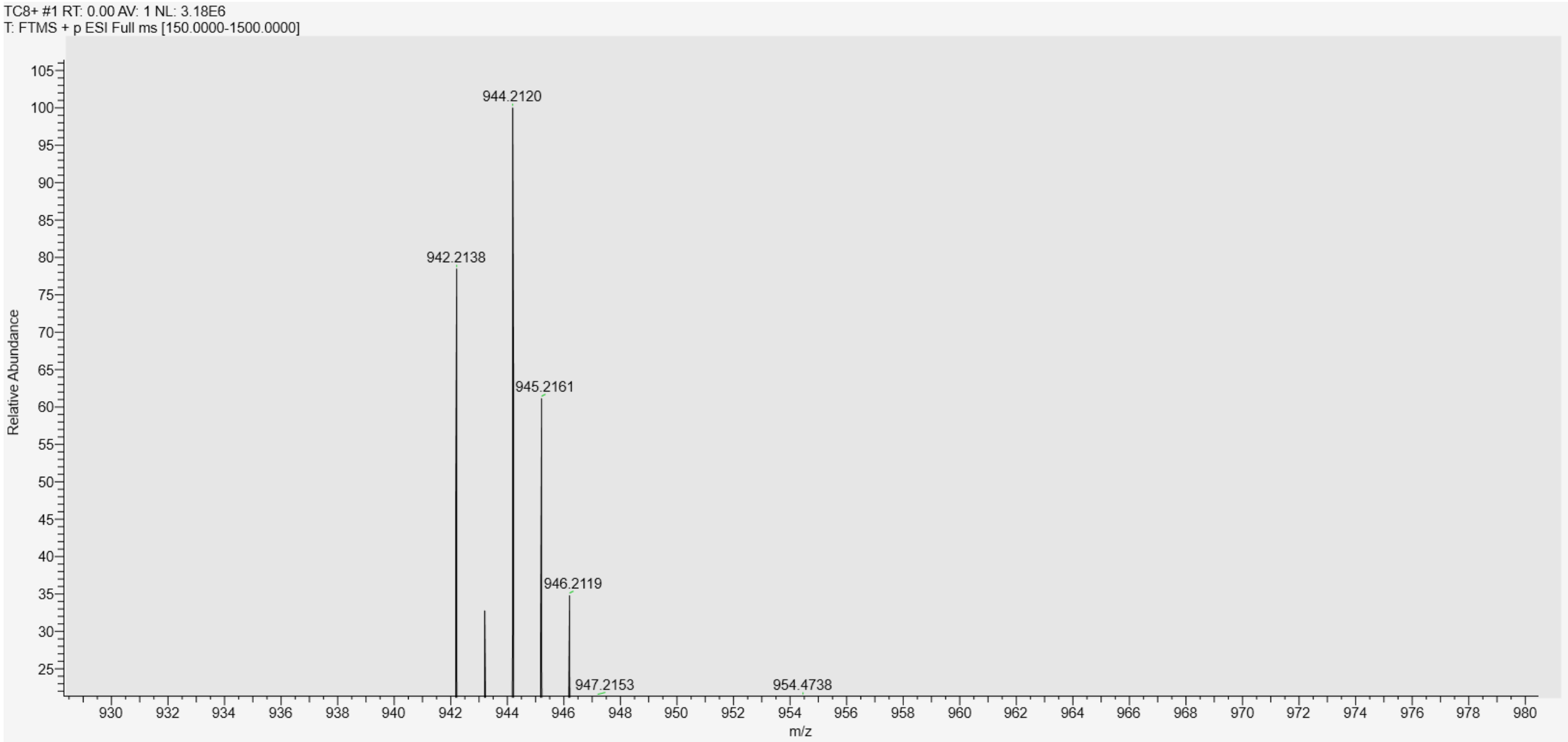

Figure S9. HRMS spectrum of TC8.

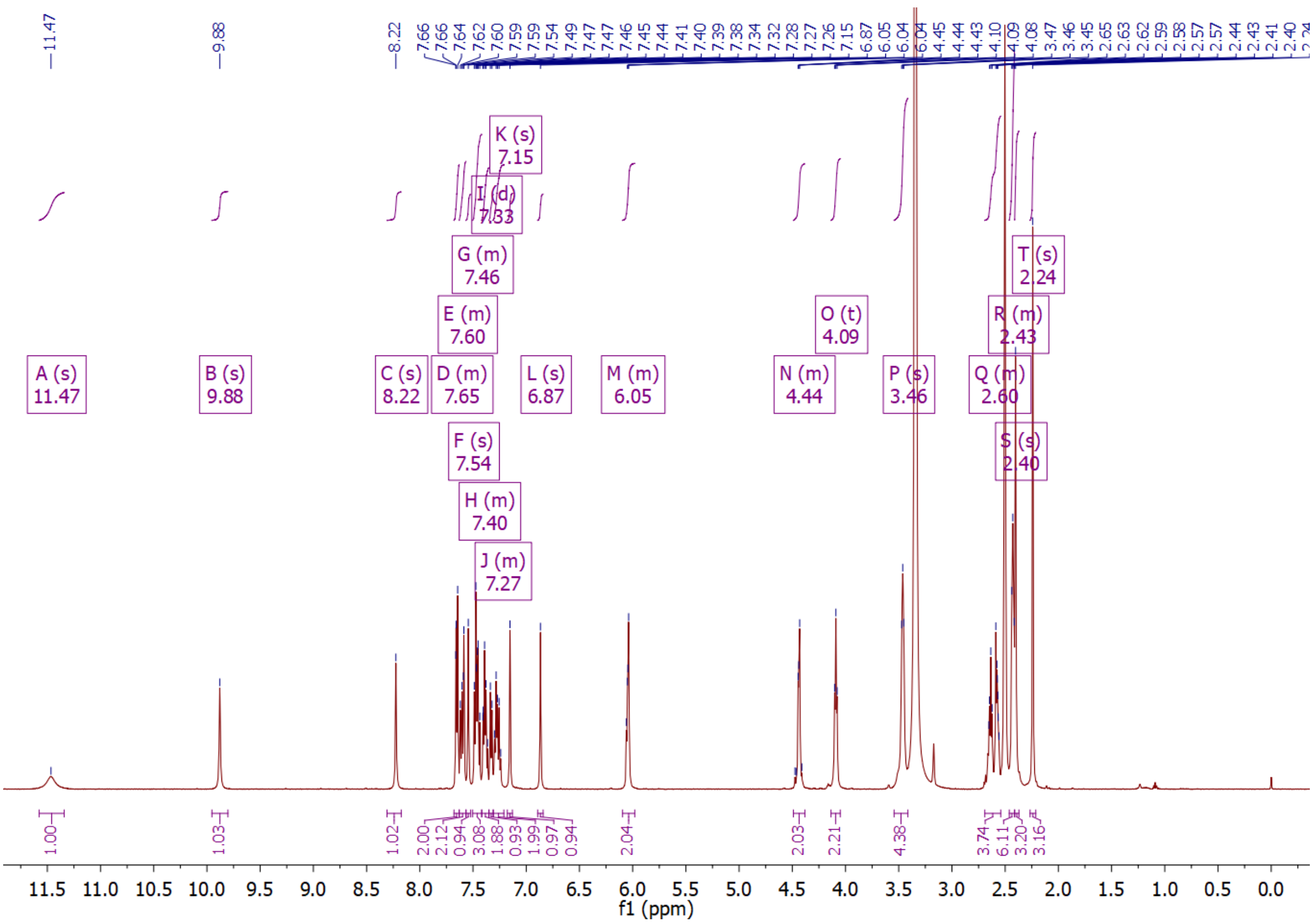

Figure S10. ^{1}H NMR (500 MHz, DMSO-d_6) spectrum of TC9.

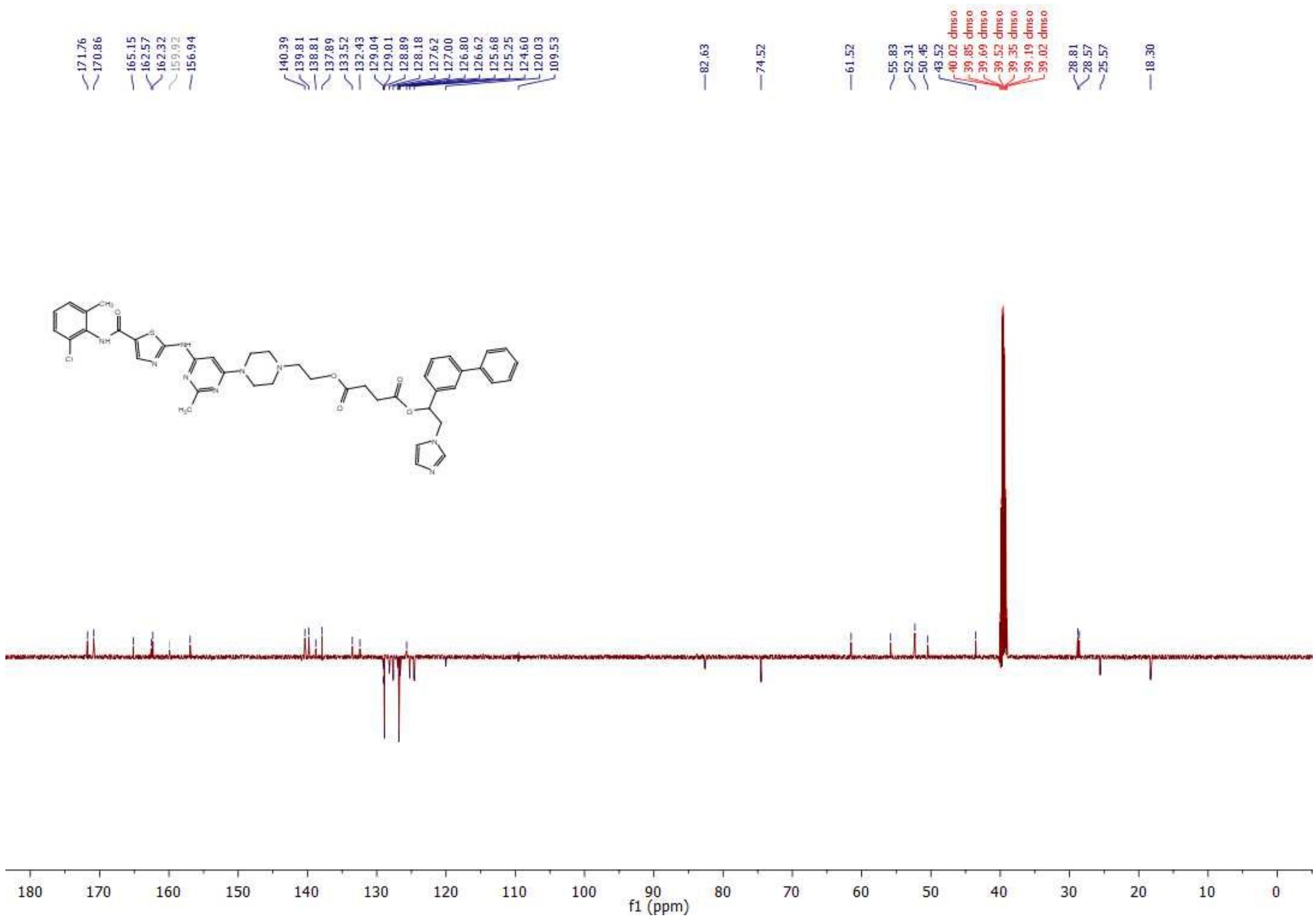

Figure S11. APT (126 MHz, DMSO-d_6) spectrum of TC9.

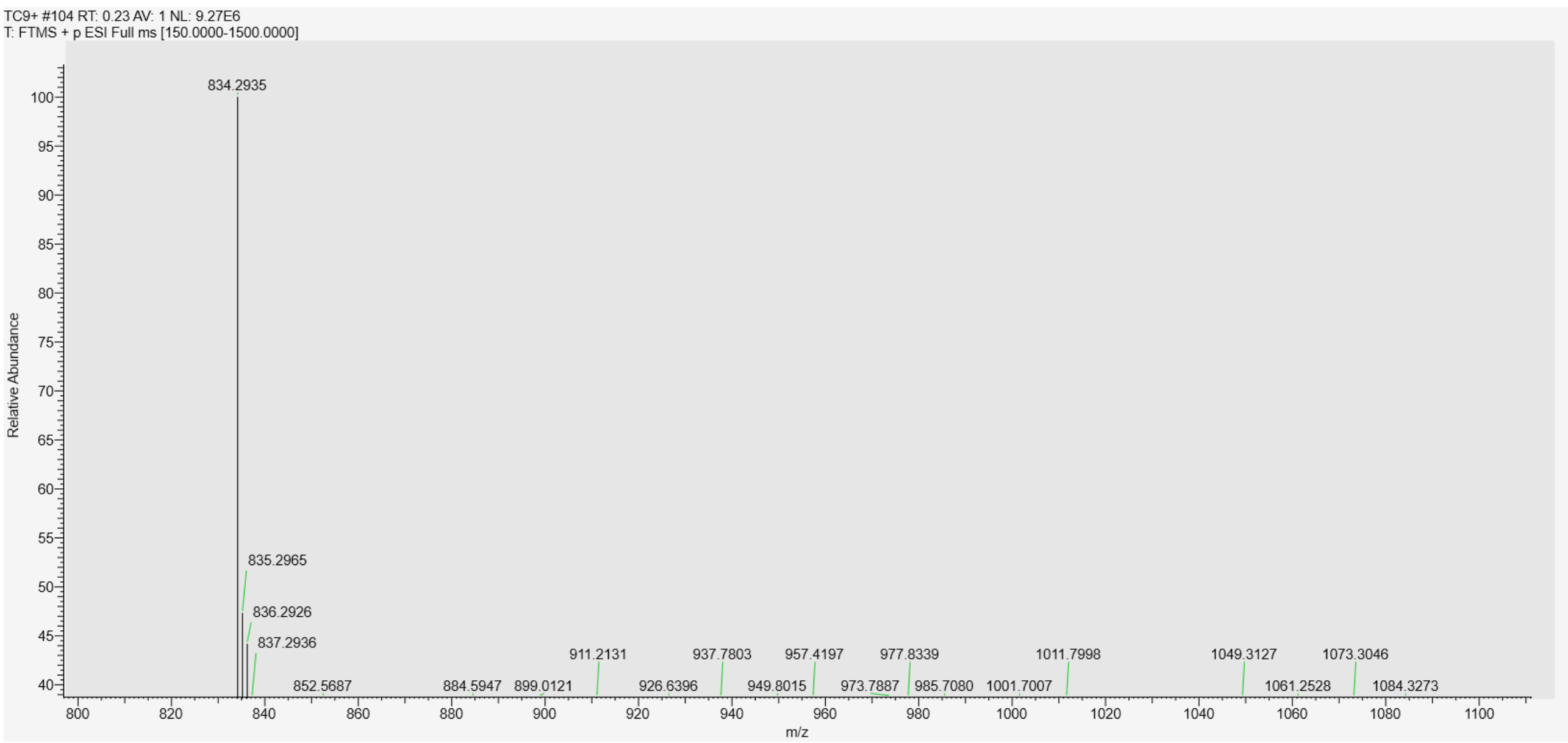

Figure S12. HRMS spectrum of TC9.

Table S1. Docking scores for selected docked poses.

**HO-1**	**Chemgauss4**	**MM-GBSA**
**TC7**	–12.749	–10.759
**TC8**	–10.927	–11.424
**TC9**	–10.092	–8.950
**cSRC**	**Chemgauss4**	**MM-GBSA**
**TC7**	–11.119	–10.022
**TC8**	–10.091	–9.745
**TC9**	–9.736	–9.423

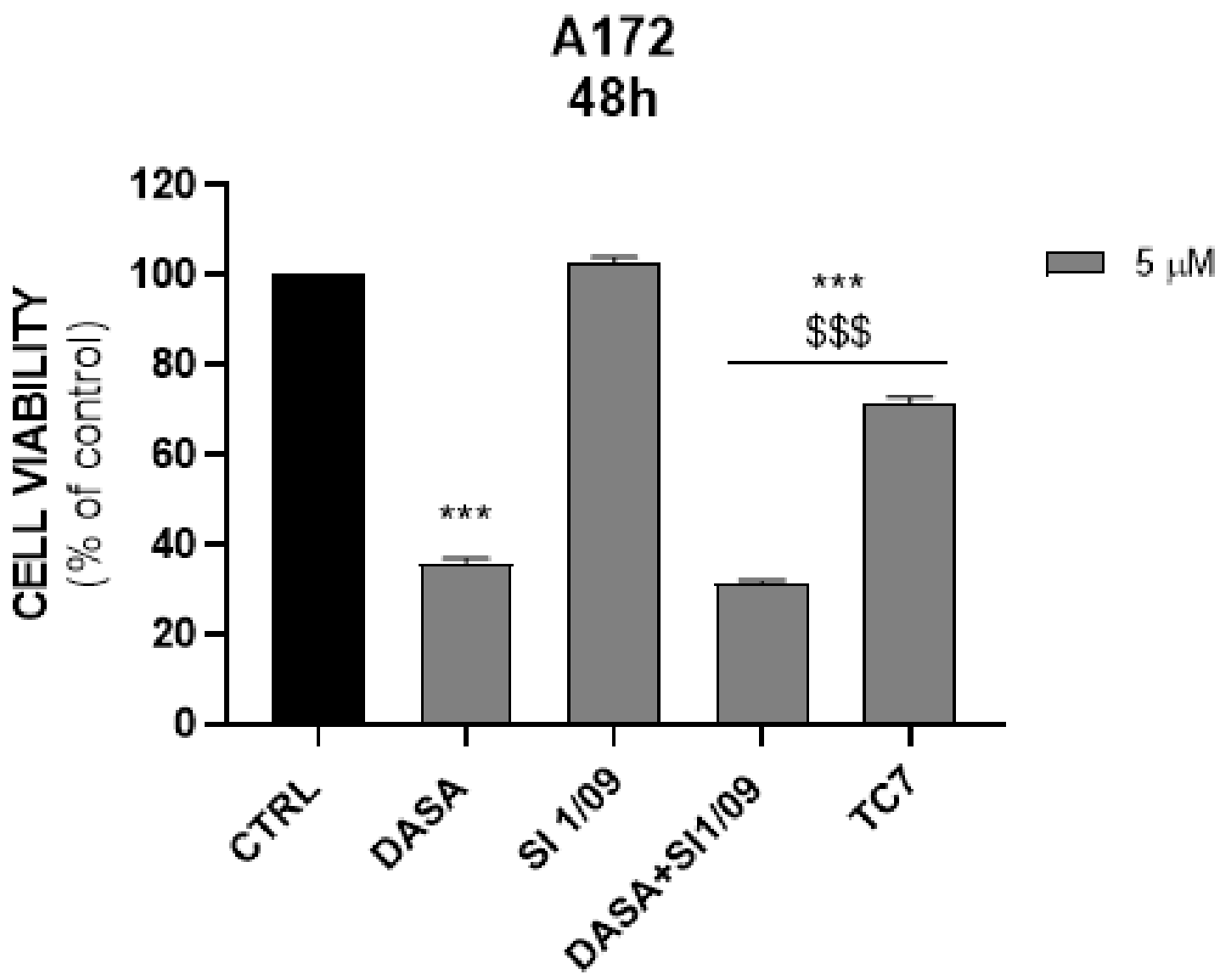

Figure S13. Effect of DASA, SI 1/09, combo, and TC7 (5 μM) on A172 cells viability at 48h. ***p<0.001 *vs* control; $$$p<0.001 *vs* SI 1/09.

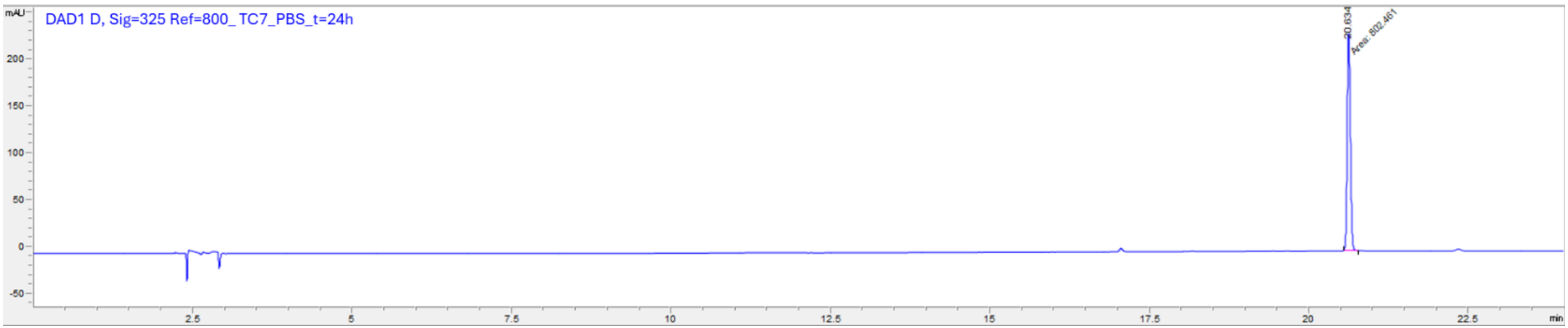

Figure S14. HPLC-chromatogram of TC7 after 24h of incubation in PBS.

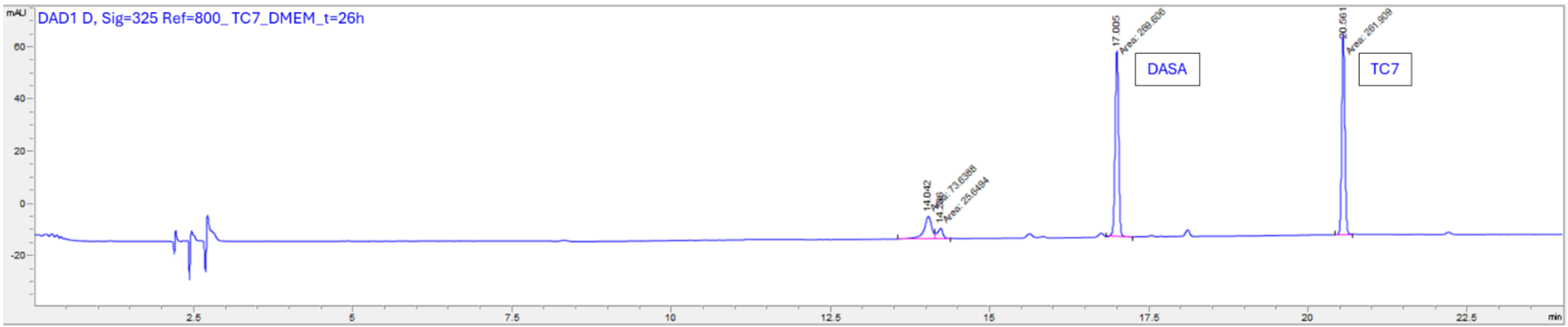

Figure S15. HPLC-chromatogram of TC7 after 26h of incubation in DMEM.

Table S2. *In vitro* absorption.

Compound I.D.	Client Compound I.D.	Test Concentration	Permeability (10^{-6} cm/s) 1st	2nd	3rd	Mean	Flags	Percent Recovery(%) 1st	2nd	3rd	Mean
**A-B permeability (MDR1-MDCKII, pH 7.4/7.4)**											
100077230-6	TC7	1.0E-05 M	0.38	0.29		<0.334	BLQ	2	1		2
**B-A permeability (MDR1-MDCKII, pH 7.4/7.4)**											
100077230-6	TC7	1.0E-05 M	0.53	0.35		<0.444	BLQ	4	1		2

BLQ: Below the Limit of Quantitation. Test compound was well detected in donor samples but not detected in receiver samples. The concentration of test compound in receiver sample was below the limit of quantitation.
